# Structural basis of regioselective tryptophan dibromination by the single-component flavin-dependent halogenase AetF

**DOI:** 10.1107/S2059798323004254

**Published:** 2023-06-14

**Authors:** Simon Gäfe, Hartmut H. Niemann

**Affiliations:** a Department of Chemistry, Bielefeld University, Universitaetsstrasse 25, 33615 Bielefeld, Germany; McGill University, Canada

**Keywords:** aetokthonotoxin, biocatalysis, bromination, enzymes, Rossmann fold, site selectivity, AetF, substrate binding

## Abstract

The flavin-dependent halogenase (FDH) AetF successively brominates tryptophan at C5 and C7 to generate 5,7-dibromotryptophan. In contrast to the well studied two-component tryptophan halogenases, AetF is a single-component flavoprotein monooxygenase. Here, crystal structures of AetF alone and in complex with various substrates are presented, representing the first experimental structures of a single-component FDH. Rotational pseudo­symmetry and pseudomerohedral twinning complicated the phasing of one structure. AetF is structurally related to flavin-dependent monooxygenases. It contains two dinucleotide-binding domains for binding the ADP moiety with unusual sequences that deviate from the consensus sequences G*X*G*XX*G and G*X*G*XX*A. A large domain tightly binds the cofactor flavin adenine dinucleotide (FAD), while the small domain responsible for binding the nicotinamide adenine dinucleotide (NADP) is unoccupied. About half of the protein forms additional structural elements containing the tryptophan binding site. FAD and tryptophan are about 16 Å apart. A tunnel between them presumably allows diffusion of the active halogenating agent hypohalous acid from FAD to the substrate. Tryptophan and 5-bromotryptophan bind to the same site but with a different binding pose. A flip of the indole moiety identically positions C5 of tryptophan and C7 of 5-bromotryptophan next to the tunnel and to catalytic residues, providing a simple explanation for the regioselectivity of the two successive halogenations. AetF can also bind 7-bromotryptophan in the same orientation as tryptophan. This opens the way for the biocatalytic production of differentially dihalogenated tryptophan derivatives. The structural conservation of a catalytic lysine suggests a way to identify novel single-component FDHs.

## Abstract 2

The single-component flavin-dependent tryptophan halogenase AetF converts tryptophan to 5,7-dibromotryptophan during the biosynthesis of the neurotoxin aetokthonotoxin. Crystal structures of AetF with the substrates tryptophan and 5-bromotryptophan show that a flip of the indole moiety of tryptophan positions first C5 and then C7 in the same location in the active site to facilitate two successive bromination reactions.

## 1. Introduction

Flavin-dependent halogenases (FDHs) mediate the regio­selective chlorination and bromination of many halogenated aromatic moieties in natural products (Neumann *et al.*, 2008 ▸; Weichold *et al.*, 2016 ▸; Agarwal *et al.*, 2017 ▸; Phintha *et al.*, 2020 ▸). Sometimes one aromatic moiety carries more than one halogen. Different routes exist for such multiple site-specific halogenations. In the biosynthesis of kutzneride, two FDHs act successively in a defined order, with 7-chlorotryptophan (7-Cl-Trp), the product of the first halogenase KtzQ, serving as the substrate of the second halogenase KtzR (Heemstra & Walsh, 2008 ▸). In other cases a single enzyme can install halogens on up to four aromatic carbons, but the structural basis of these multiple halogenations often remains elusive (Dorrestein *et al.*, 2005 ▸; Pang *et al.*, 2015 ▸; El Gamal *et al.*, 2016 ▸). The mechanism of the FDH MalA is exceptionally well understood. During the biosynthesis of malbrancheamides, MalA regioselectively halogenates two positions of the substrate. There is no defined order and the product of the first halogenation does not need to reorient in the enzyme for the second halogenation to occur (Fraley *et al.*, 2017 ▸).

Aetokthonotoxin (AETX) is a pentabrominated natural compound with two indole moieties, one of which carries two bromines and the other three. AETX was recently discovered as a cyanobacterial toxin that kills bald eagles and other animals in the USA (Breinlinger *et al.*, 2021 ▸). The gene cluster for AETX biosynthesis encodes the FDHs AetA and AetF. AetF converts tryptophan to 5-bromotryptophan (5-Br-Trp) and 5-Br-Trp to 5,7-dibromotryptophan (5,7-Br_2_-Trp) (Breinlinger *et al.*, 2021 ▸). AetA brominates 5-bromoindole (5-Br-indole) to 3,5-dibromoindole (3,5-Br_2_-indole), which it then converts to the final product 2,3,5-tribromoindole (2,3,5-Br_3_-indole) (Adak *et al.*, 2022 ▸). AetA requires a separate flavin reductase to regenerate the reduced cofactor FADH_2_. It thus belongs to the two-component FDHs, which constitute the majority of currently known FDHs, including the well characterized tryptophan halogenases (Dong *et al.*, 2005 ▸; Dachwitz *et al.*, 2021 ▸). AetF is a single-component FDH that uses NADPH to reduce FAD (Adak *et al.*, 2022 ▸). AetF is more closely related to single-component flavin-dependent monooxygenases than to any of the other characterized flavin-dependent tryptophan halogenases (Adak *et al.*, 2022 ▸; Paul *et al.*, 2021 ▸). The phenol brominase Bmp5 has previously been described as a single-component FDH (Agarwal *et al.*, 2014 ▸). However, single-component FDHs remain poorly characterized in terms of reaction mechanism, substrate binding and regioselectivity. Docking of the substrate into an *AlphaFold*2 model of AetF allowed the identification of a catalytically essential lysine residue but provided no clear explanation for the regioselective dibromination of tryptophan (Jiang *et al.*, 2022 ▸).

Here, we set out to unravel the structural basis of regio­selective dibromination by AetF. We determined crystal structures of AetF, including structures with the two natural substrates tryptophan and 5-Br-Trp. These structures facilitate a better understanding of single-component FDHs in general.

## 2. Methods

### 2.1. Protein expression

The *Escherichia coli* codon-optimized C-terminally truncated variant *aetF*_1–655_ was synthesized and subcloned into a pET-24(+) vector between the BamHI and XhoI restriction sites by Twist Bioscience. The synthesized insert additionally contained an N-terminal His_6_ tag and Tobacco etch virus (TEV) protease cleavage site. The plasmid was transformed into *E. coli* BL21(DE3) cells via heat shock. An overnight preculture (37°C, 120 rev min^−1^) grown in LB medium (30 µg ml^−1^ kanamycin) was used to inoculate 2 l TB medium (30 µg ml^−1^ kanamycin) to an optical density at 600 nm (OD_600_) of 0.1. The culture was incubated at 37°C and 90 rev min^−1^ until it reached an OD_600_ of 0.4, cooled to 18°C and expression was induced at an OD_600_ of 0.6–0.8 by adding 0.1 m*M* isopropyl β-d-1-thiogalactopyranoside (IPTG). The induced cultures were incubated for 20 h at 18°C and 90 rev min^−1^ before harvesting the cells via centrifugation (4500*g*, 30 min, 4°C). The cell pellets were washed with cooled phosphate-buffered saline (PBS) and frozen at −20°C.

### 2.2. Protein purification

The pellet from 2 l TB culture was resuspended in ∼70 ml cold lysis buffer (50 m*M* HEPES pH 7, 500 m*M* NaCl, 20 m*M* imidazole pH 7, 10 m*M* β-mercaptoethanol) and lysed via a SPCH ultrahigh-pressure cell homogenizer (Stansted Fluid Power) in three cycles at 1.17 MPa. Soluble protein was separated from insoluble compounds by centrifugation (30 000*g*, 30 min, 4°C). The supernatant was incubated with Co^2+^–NTA Agarose (Cube Biotech) for 60 min at 4°C. After washing with lysis buffer, the protein was eluted (50 m*M* HEPES pH 7, 500 m*M* NaCl, 250 m*M* imidazole pH 7, 10 m*M* β-mercaptoethanol) in 5 ml fractions and 5 m*M* EDTA was added immediately afterwards to reduce protein aggregation. His_6_-TEV-AetF_1–655_-containing fractions were pooled, concentrated (Pierce, 10 000 Da molecular-weight cutoff) and desalted using PD-10 desalting columns (GE Healthcare) equilibrated with size-exclusion chromatography (SEC) buffer (50 m*M* HEPES pH 7, 100 m*M* NaCl, 1 m*M* DTT). In order to remove the His_6_ tag, TEV protease (1 mg TEV protease per 50 mg protein) was added and incubated overnight at 20°C. In a subsequent Co^2+^–NTA chromatography, the cleaved AetF_1–655_ was obtained in the flowthrough and washing fractions and was concentrated (Pierce, 10 000 Da molecular-weight cutoff). SEC was performed at 4°C with SEC buffer using a 16/60 Superdex 200 column (GE Healthcare) connected to an ÄKTA purifier system. The eluted protein was concentrated to 19.19 mg ml^−1^ and stored at −80°C. The protein had a bright yellow colour in all purification steps, indicating high FAD occupancy. UV–Vis spectroscopy (NanoDrop 1000, PEQLAB) confirmed an occupancy of close to 100% for FAD.

### 2.3. Crystallization

AetF_1–655_ was diluted to 6 mg ml^−1^ with SEC buffer. Initial crystallization trials comprised eight commercial screens at 4 and 20°C in MRC 2-Lens sitting-drop plates (SWISSCI) with two drop ratios (300 nl protein solution + 300 nl reservoir solution and 600 nl protein solution + 300 nl reservoir solution). Yellow spear tip-shaped crystals appeared within 2–4 days in ∼6% of the tested conditions. The crystals were harvested after 4–5 days. The best diffracting crystals grew at 4°C in condition A6 [30 m*M* CaCl_2_, 30 m*M* MgCl_2_, 0.1 *M* HEPES/MOPS pH 7.5, 20%(*v*/*v*) ethylene glycol, 10%(*w*/*v*) PEG 8000] of the Morpheus screen (Molecular Dimensions) and yielded the non-substrate-bound structure (empty AetF). AetF_1–655_ (6 mg ml^−1^) was co-crystallized with substrates under the same conditions and temperature. Instead of mixing protein and substrate beforehand, we dried 300 nl of 40 m*M* l-Trp (AppliChem GmbH), saturated 5-Br-Trp (Chem-Impex) or saturated 7-bromotryptophan (7-Br-Trp; provided by Nicolai Montua, OC III, Bielefeld University) on the plate prior to setting up the crystallization plate as described above. This resulted in 5-Br-Trp-bound AetF_1–655_ co-crystals with the same colour and morphology, but did not yield co-crystals with l-Trp or 7-Br-Trp. Therefore, l-Trp and 7-Br-Trp-bound AetF_1–655_ crystals were obtained by soaking non-substrate-bound crystals with l-Trp [250 nl 37.5 m*M* l-Trp, 25%(*v*/*v*) ethylene glycol] and 7-Br-Trp [150 nl sat. 7-Br-Trp, 25%(*v*/*v*)], respectively. All crystals were flash-cooled in liquid nitrogen without additional cryoprotectant.

### 2.4. Data collection and structure determination

X-ray diffraction data for the non-substrate-bound crystals (empty AetF) were collected at a wavelength of 0.97626 Å on beamline P13 operated by EMBL Hamburg at the PETRA III storage ring, DESY, Hamburg, Germany (Cianci *et al.*, 2017 ▸). Measurements of 5-Br-Trp co-crystals and crystals soaked with l-Trp were carried out at a wavelength of 0.9184 Å on beamline ID30B (McCarthy *et al.*, 2018 ▸) at the European Synchrotron Radiation Facility (ESRF), Grenoble, France. Data for AetF_1–655_ soaked with 7-Br-Trp (measured at 0.88560 Å) were collected on beamline ID23-1 (Nurizzo *et al.*, 2006 ▸) at the ESRF. All data were collected at 100 K and the beamlines were controlled using *MxCuBE* (Gabadinho *et al.*, 2010 ▸; Mueller *et al.*, 2017 ▸). Diffraction data were indexed, integrated and scaled using *XDS* (Kabsch, 2010 ▸), treating Friedel pairs as different reflections for the bromine-containing structures. The structure of empty AetF was solved by molecular replacement (MR) via *Phaser* (McCoy *et al.*, 2007 ▸) using a truncated model generated by a local installation of *AlphaFold*2 (Jumper *et al.*, 2021 ▸). The final version of empty AetF was used to solve the isomorphous 5-Br-Trp-bound structure (AetF–5-Br-Trp) by rigid-body refinement against the respective data in *phenix.refine* (Afonine *et al.*, 2012 ▸; Liebschner *et al.*, 2019 ▸). The same model was used to solve the l-Trp-bound and 7-Br-Trp-bound structures (AetF–Trp and AetF–7-Br-Trp) via MR by searching for four molecules per asymmetric unit in space group *P*2_1_2_1_2_1_ for AetF–Trp and two molecules per asymmetric unit in space group *P*4_3_ for AetF–7-Br-Trp. All models were optimized by several rounds of model building in *Coot* (Emsley *et al.*, 2010 ▸) and refinement using *phenix.refine* (Afonine *et al.*, 2012 ▸; Liebschner *et al.*, 2019 ▸). Different refinement strategies were tested and the best *R*_free_ values were obtained by refining atomic coordinates, individual *B* factors and occupancies using TLS parameters (generated by *phenix.refine*) and noncrystallographic symmetry (NCS) restraints and by optimizing both X-ray/stereochemistry and X-ray/ADP weights. H atoms were automatically added for the empty AetF structure and the complex with 5-Br-Trp. For the 5-Br-Trp-bound and 7-Br-Trp-bound structures (AetF–5-Br-Trp and AetF–7-Br-Trp) the Br atoms were refined as anomalous groups. The twin laws −*k*, −*h*, −*l* for the twinned AetF–7-Br-Trp structure (solved in space group *P*4_3_) and −*h*, −*l*, −*k* for the twinned AetF–Trp structure (solved in space group *P*2_1_2_1_2_1_) were determined via twin refinement in *REFMAC*5 (Kovalevskiy *et al.*, 2018 ▸) and were used in all refinements. For all four structures, the resolution limit was determined with paired refinement (Karplus & Diederichs, 2012 ▸) as implemented in *PAIREF* (Malý *et al.*, 2020 ▸). Data-collection and refinement statistics are summarized in Tables 1 ▸ and 2 ▸. All structural illustrations were generated in *PyMOL*. The coordinates and structure factors of the final models were deposited in the Protein Data Bank with accession codes 8cjd (empty AetF), 8cje (AetF–Trp), 8cjf (AetF–5-Br-Trp) and 8cjg (AetF–7-Br-Trp). Diffraction images are available via the SBGrid Data Bank (Meyer *et al.*, 2016 ▸) and additionally via the ESRF Data Portal for data collected at the ESRF. DOIs for the data sets are given in Table 1 ▸.

## 3. Results and discussion

### 3.1. Construct design

Initially, we worked with full-length AetF expressed as His_6_-TEV-AetF with a single alanine inserted between the TEV protease site and AetF, as found in pETM11 vectors. TEV protease digestion of this construct turned out to be impossible, although this short linker between the TEV site and the protein of interest had worked for many other proteins. This construct resulted in irreproducible crystals that diffracted to about 3 Å resolution. Attempts to solve the structure by molecular replacement failed. When an *AlphaFold*2 model became available, we modified the construct in two ways. Firstly, we noticed that the start methionine hardly protrudes from the well folded core, resulting in steric hindrance of TEV protease, preventing it from binding and cutting at its recognition site. Insertion of an additional (SG)_3_ linker solved this problem and resulted in complete TEV protease digestion. Secondly, the *AlphaFold*2 model suggested that the C-terminus was unfolded, which could impair crystallization. Therefore, we deleted the last 13 amino acids, resulting in the His_6_-TEV-AetF_1–655_ construct described in this work. TEV protease-digested and purified AetF_1–655_ (hereafter referred to as AetF) exhibited exceptionally good crystallization properties, yielding crystals in 99 of the 1536 conditions tested. We collected data from crystals grown in ten different conditions. All tested crystals had the same unit-cell dimensions, suggesting that they have the same packing. We settled on condition A6 of the Morpheus screen (Gorrec, 2009 ▸) because these crystals showed the best diffraction. This illustrates how *AlphaFold*2 models can help to streamline construct design for crystallization.

### 3.2. Structure determination from untwinned and twinned crystals in space groups *P*4_3_ and *P*2_1_2_1_2_1_ both exhibiting pseudo-*P*4_3_2_1_2 symmetry

**Figure 1:**
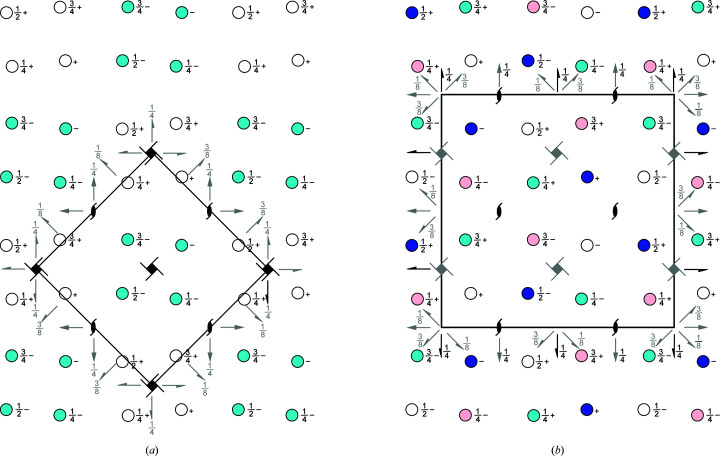
Pseudo-*P*4_3_2_1_2 packing of both AetF crystal forms. Crystallographic and noncrystallographic symmetry elements are shown in black and grey, respectively. Crystallographically independent molecules are shown in different colours. (*a*) *P*4_3_ packing with two molecules per asymmetric unit. (*b*) *P*2_1_2_1_2_1_ packing with four molecules per asymmetric unit.

Structure determination was straightforward for non-substrate-bound (empty AetF) and 5-Br-Trp-bound AetF (AetF–5-Br-Trp). These crystals were isomorphous to each other and scaled in space groups *P*4_1_ or *P*4_3_ without signs of twinning or translational noncrystallographic symmetry (tNCS). Empty AetF was solved in space group *P*4_3_ by molecular replacement with two molecules per asymmetric unit. The structure of AetF co-crystallized with 5-Br-Trp was phased by the difference Fourier method. The data for AetF soaked with 7-Br-Trp scaled reasonably well in point group 422. These crystals turned out to be isomorphous to the non-substrate-bound and the 5-Br-Trp crystals, but were twinned with twin operator −*k*, −*h*, −*l* and a twin fraction of 20%. All data sets for AetF soaked with Trp scaled well in point group 422 and showed a larger unit cell with a roughly doubled volume. Molecular replacement searching for two molecules per asymmetric unit in all possible space groups of point group 422 gave two solutions in space group *P*4_3_2_1_2 with TFZ values of 19.2 for the first chain in both solutions and 17.2 and 15.3 for the second chain of the respective solutions. However, there were severe clashes and the electron density was poor. A closer inspection of the data suggested a combination of twinning and tNCS. The length of the tetragonal unit-cell *c* axis of Trp-bound AetF is roughly the same compared with the other AetF structures, while the *a* and *b* axes are roughly 1.4 (the square root of 2) times longer. *phenix.xtriage* revealed a small tNCS peak at 0.484, 0.489, 0.0 with a height of 12.3% of the origin peak. The cumulative intensity distribution from *AIMLESS* and the *L*-test in *phenix.xtriage* both indicated a high degree of twinning. Therefore, we assumed a pseudo-*C*-centred cell, with the real space group being *P*4_3_, and a twofold twin operator around *a*. Molecular replacement searching for four molecules in *P*4_3_ produced four solutions, which only differed in the placement of the fourth chain. The TFZ values were 44.1, 32.3 and 52.6 for first three chains and 7.4, 7.1, 5.5 and 5.1 for the four different solutions of the fourth chain. The packing produced severe clashes and the electron density was very poor for two of the chains. Subsequent inspection of individual symmetry operators showed that a fourfold axis along *c* had slightly worse *R*_meas_ and *I*/σ(*I*) values than each of the three twofold axes along *a*, *b* and *c*, suggesting that the true point group could be 222 with a twin operator emulating a fourfold axis, which is visible in the self-rotation functions (Supplementary Fig. S1). Molecular replacement searching for four molecules per asymmetric unit in all possible space groups of point group 222 resulted in a solution in *P*2_1_2_1_2 (TFZ = 38.9), but again the electron density was poor. Explicitly searching in *P*2_1_2_1_2_1_ gave a unique solution for four molecules per asymmetric unit with TFZ values of 19.5, 39.3, 41.1 and 101.6. Refinement without any rebuilding resulted in *R*_work_ = 29.40%, *R*_free_ = 34.64% without twin refinement and *R*_work_ = 19.71%, *R*_free_ = 22.07% with twin refinement, suggesting *P*2_1_2_1_2_1_ to be the correct space group. To scrutinize our choice of space group, we solved the structure in *P*1. *Phaser* managed to place 15 of the 16 molecules and we manually placed the 16th molecule. Based on the *P*1 structure, *Zanuda* (Lebedev & Isupov, 2014 ▸) confirmed *P*2_1_2_1_2_1_ to be the most likely space group. The refined twin fraction for twin operator −*h*, −*l*, −*k* is 45%. The packing of AetF is very similar in the small *P*4_3_ and the larger *P*2_1_2_1_2_1_ unit cells. In *P*4_3_ there are noncrystallographic twofold and 2_1_ axes, resulting in a pseudo-*P*4_3_2_1_2 packing (Fig. 1 ▸ *a*). *P*2_1_2_1_2_1_ is a *translationsgleiche* subgroup of *P*4_3_2_1_2. In the larger *P*2_1_2_1_2_1_ unit cell, there are noncrystallographic twofold, 2_1_ and 4_3_ axes, again resulting in a pseudo-*P*4_3_2_1_2 packing (Fig. 1 ▸ *b*).

### 3.3. Only about half of AetF forms the two conserved nucleotide-binding domains, while the additional structural elements contain a catalytic lysine

AetF eluted as monomer from gel filtration and it is monomeric in the crystalline state. In all solved structures the complete sequence could be built except for the N-terminal (SG)_3_ linker and a disordered loop (residues 608 ± 2 to 630 ± 2). AetF retained a tightly bound FAD during purification and FAD is clearly visible in all chains. Refinement yielded occupancies of ∼1 for all FADs.

The *AlphaFold*2 model of AetF employed as a search model for MR represents a good structural model with an overall r.m.s.d. of 1 Å for 632 aligned C^α^ atoms. A *DALI* search (Holm, 2022 ▸) with our structure (Supplementary Table S1) returned many good hits with *Z*-scores above 20, including several ancestral flavin-containing monoxygenases (AncFMO2, AncFMO5, AncFMO3 and AncFMO1; Nicoll *et al.*, 2020 ▸; Bailleul *et al.*, 2021 ▸) and many other flavoprotein monooxygenases (FPMOs) such as pyrrolizidine alkaloid *N*-oxygenase from *Zonocerus variegatus* (*Zv*PNO; Kubitza *et al.*, 2018 ▸), phenylacetone monooxygenase (Malito *et al.*, 2004 ▸) and cyclohexanone monooxygenase (Mirza *et al.*, 2009 ▸). While the latter two are Baeyer–Villiger monooxygenases and belong to FMPO subgroup B1, the structurally most similar enzymes and Bmp5, the only other known single-component FDH, belong to FMPO subgroup B2 (Paul *et al.*, 2021 ▸), suggesting that AetF can also be assigned to subgroup B2 and therefore is a flavin-dependent monooxygenase (FMO). Because there is no experimental structure of Bmp5, we overlaid our AetF structure with the *AlphaFold*2 model of Bmp5 (Varadi *et al.*, 2022 ▸). A pairwise alignment with the *DALI* server gave a *Z*-score of 27.6, an r.m.s.d. of 3.5 Å for 438 aligned residues and a sequence identity of 16% (Supplementary Table S1).

**Figure 2:**
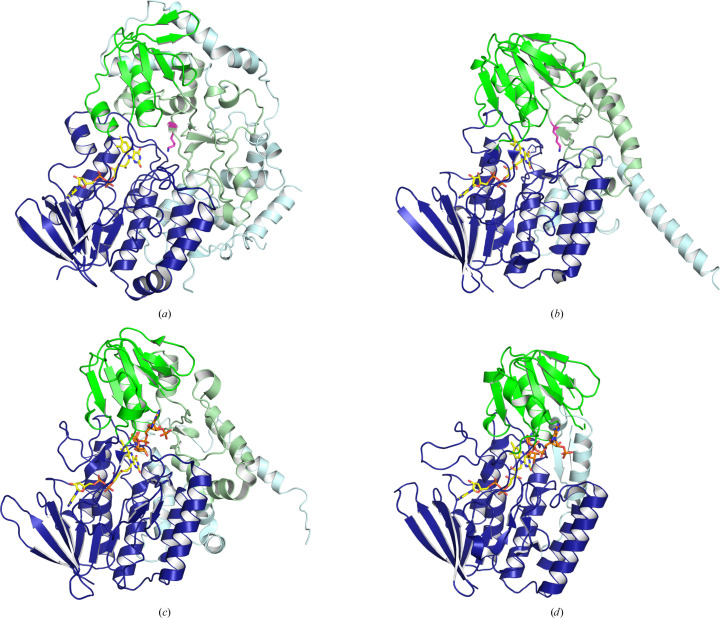
Domain organization of AetF and structurally related FMOs. The FAD- and NADP-binding domains are shown in dark blue and green, respectively. Additional structural elements inserted into the NADP-binding domain or C-terminally appended to the FAD-binding domain are shown in light green and light blue, respectively. The catalytic lysines of AetF (Lys258) and Bmp5 (Lys277) are shown as stick models with pink C atoms. FAD and NAPD are shown with yellow and orange C atoms, respectively. The domain borders used in this figure are given in Supplementary Table S2. (*a*) AetF (PDB entry 8cjd). (*b*) Bmp5 [*AlphaFold2* model with FAD from PDB entry 6sek optimized by *AlphaFill* (Hekkelman *et al.*, 2023 ▸), AF-U6BHD3-F1-model_v4-B-optimized]. (*c*) AncFMO2 (PDB entry 6sf0). (*d*) *Zv*PNO (PDB entry 5nmx).

Group B FPMOs contain two domains with dinucleotide-binding Rossmann folds: a larger FAD-binding domain and a smaller NADP-binding domain (Paul *et al.*, 2021 ▸). Both domains are present in AetF. Although we use the term domains here, the overall structure and the distribution of hydrophobic residues suggest that it might be more fitting to describe AetF as single-domain protein with several sub­domains. A structural comparison with other FMOs such as Bmp5 and the ancestral AncFMO2 (*DALI* *Z*-score 26.5; PDB entry 6sf0) and with *Zv*PNO (*Z*-score 18.3; PDB entry 5nmx) is complicated by the fact that the dinucleotide-binding domains are not contiguous in sequence except for the small domain of *Zv*PNO. The small NADPH-binding domain is inserted into the large FAD-binding domain. In AetF, Bmp5 and the ancestral FMOs, the small domain is also split, containing an insertion that forms additional structural elements together with the C-terminus. In AetF, these extra structures comprise about half of the sequence (Fig. 2 ▸). Some structural elements that we assign to the insertion in the small domain [a β-strand (residues 177–184) and a β-strand followed by a short helix (residues 257–293)] are conserved between AetF and Bmp5 and may eventually be assigned to the small domain instead. In the C-terminal extension, the sequence 469-YRL*X*G-473 is conserved between AetF, Bmp5 and AncFMO2, with the tyrosine and arginine forming extensive conserved interactions with the large domain via hydrogen bonds and salt bridges, supporting our assumption that the whole protein may fold as a single unit.

**Figure 3:**
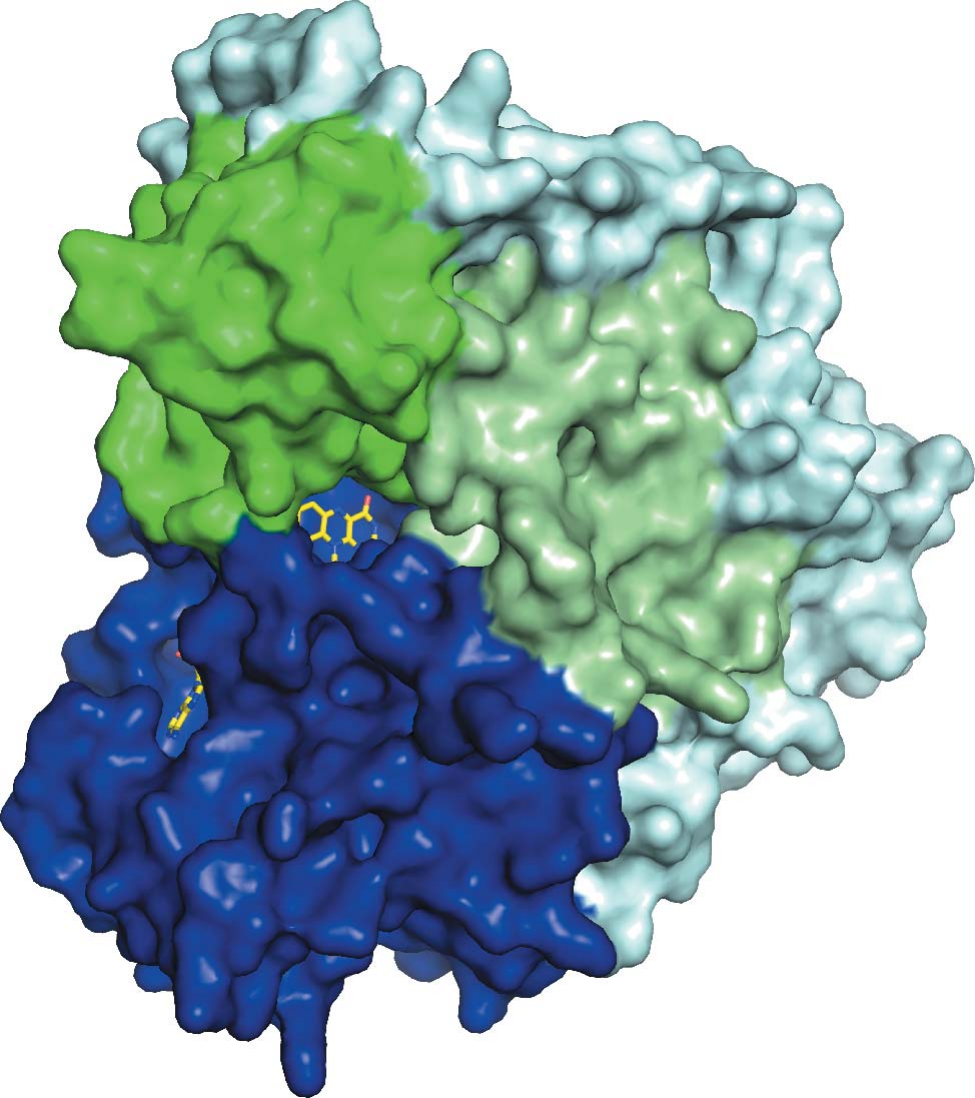
Surface representation of AetF with FAD shown as a stick model. Domains are coloured as in Fig. 2 ▸.

AetF almost completely envelops the FAD, which explains the tight binding and high occupancy of FAD without any addition of cofactor (Fig. 3 ▸). The consensus sequences for dinucleotide binding in Rossmann folds are G*X*G*XX*G for FAD and pyridine dinucleotides (as found, for example, in *Zv*PNO) and sometimes G*X*G*XX*A for NADP (as found, for example, in AncFMO2) (Hanukoglu, 2015 ▸). In AetF these sequences deviate from the consensus, with 8-GFGFSA-13 for FAD and 154-TMGDSA-159 for NADP (Supplementary Fig. S2). Our AetF structure shows a large empty space where NADP is bound in *Zv*PNO and AncFMO2. Without the addition of NADP, significant difference density at the expected NADP binding site is visible, especially in the structure of AetF–5-Br-Trp, which could indicate NADP bound with low occupancy (Supplementary Fig. S3). However, this electron density is not sufficient to reliably model an NADP or even only a nicotinamide ribose monophosphate. Co-crystallization with NADP under the same conditions did not result in any crystals and soaking our crystals with NADP did not improve the electron density for the presumed NADP. This could be due to crystal packing, as the adenine of NADP from a structurally aligned AncFMO2 would clash with a symmetry mate of AetF in our AetF crystals. Hence, we assume that the reduction of FAD proceeds analogously to that in other FMOs by binding NADPH in this region. Thereby, the tightly bound FAD can be reduced without having to leave the protein.

All known two-component FDHs have a strictly conserved catalytic lysine in the active site that is most to likely to serve to polarize and activate hypohalous acid (Dong *et al.*, 2005 ▸; Flecks *et al.*, 2008 ▸; Andorfer *et al.*, 2022 ▸). Based on docking tryptophan into the active site next to FAD in an *AlphaFold*2 model of AetF, Lewis and coworkers found the 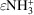 group of Lys258 to be located next to the substrate. Mutational analysis showed Lys258 to be essential for catalysis (Jiang *et al.*, 2022 ▸). The catalytic lysine of AetF is located in the insertion within the NADP-binding domain in the region that is structurally similar in AetF and Bmp5. Lysine at this position is not generally conserved among FMOs. It is not present in the ancestral FMOs and *Zv*PNO (Nicoll *et al.*, 2020 ▸; Bailleul *et al.*, 2021 ▸; Kubitza *et al.*, 2018 ▸). Interestingly, the phenol halogenase Bmp5 has an equivalent lysine, suggesting that Lys258 of AetF and Lys277 of Bmp5 may fulfil a similar function to that of the catalytic lysine in two-component FDHs.

In an attempt to locate the catalytic halide binding site, we co-crystallized AetF with Br^−^ up to a concentration of 200 m*M*. Data sets collected at the bromine absorption edge did not show strong anomalous signal and there was no anomalous difference density for bound bromide. It is conceivable that either the binding of NADP(H) or the reduction of FAD to FADH_2_ is required to form a functional bromide binding site in AetF. Further experiments will be necessary to settle this issue.

### 3.4. Tryptophan and 5-Br-Trp bind to the same site with a flipped indole moiety

**Figure 4:**
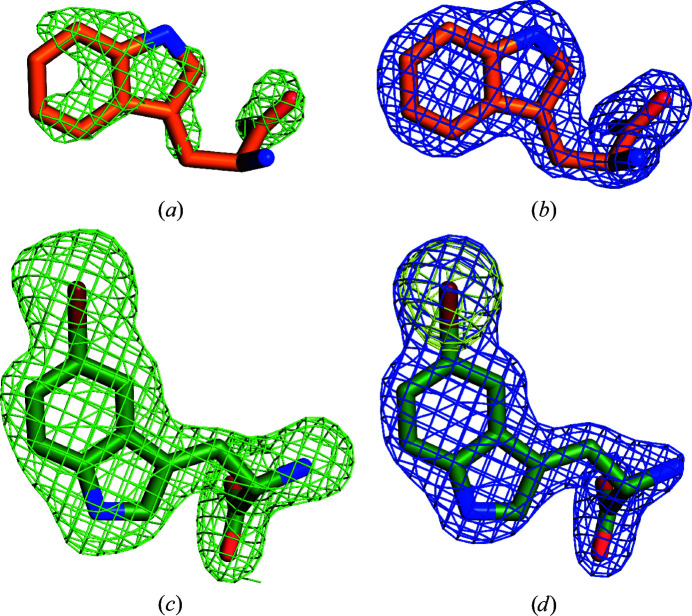
Electron density of bound substrates. Initial *mF*_o_ − *DF*_c_ density before placing the ligand is shown as a green mesh at 3σ and the final 2*mF*_o_ − *DF*_c_ density is shown as a blue mesh at 1.5σ. (*a*, *b*) Bound Trp from PDB entry 8cje chain *C*. (*c*, *d*) Bound 5-Br-Trp from PDB entry 8cjf chain *A*. The anomalous signal of bromine in 5-Br-Trp is shown as a yellow mesh (5σ).

Soaking AetF crystals with Trp and co-crystallization with 5-Br-Trp provided highly interpretable difference densities by the difference Fourier method or by MR, showing unique binding positions. After incorporation of the respective ligands, the occupancies refined to greater than 90%, except for one Trp with an occupancy of 65% (Fig. 4 ▸). For each ligand, there is no evidence of alternative binding poses in addition to the pose we modelled and the binding poses are conserved in all chains of the same structure. In the case of 5-Br-Trp, the position of the Br atom could be unambiguously determined based on the anomalous difference density.

**Figure 5:**
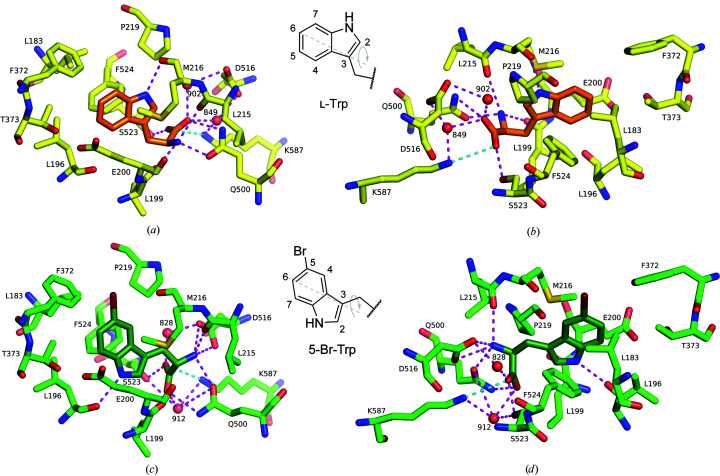
Substrate binding shown in two views. Polar contacts are shown as magenta dashed lines. A salt bridge between Lys587 and the substrate with a distance of longer than 3.5 Å is shown as a cyan dashed line. Only waters connecting the ligand and protein are shown. (*a*, *b*) Tryptophan (orange C atoms) bound to AetF (yellow C atoms) from PDB entry 8cje chain *C*. (*c*, *d*) 5-Br-Trp (dark green C atoms) bound to AetF (green C atoms) from PDB entry 8cjf chain *A*. Schematic representations of the indole moieties of tryptophan and 5-bromotryptophan with numbered C atoms are added. The grey dashed lines indicate the rotational axis between the binding poses of both ligands.

As in two-component tryptophan halogenases (Dong *et al.*, 2005 ▸; Moritzer *et al.*, 2019 ▸), the indole moiety of Trp is mostly surrounded by hydrophobic and aromatic residues (for example Leu183, Leu196, Met216, Pro219, Phe372 and Phe524; Figs. 5 ▸ *a* and 5 ▸ *b*). The indole binding site is completed by the side chains of Glu200 and Thr373. The NH group of indole forms a hydrogen bond to the carbonyl of Met216. Overall, the indole binding site appears to be rather spacious. The backbone atoms of Trp are positioned by direct and water-mediated polar contacts with surrounding amino acids. The amino group of Trp forms hydrogen bonds to the side-chain O atom of Gln500 and the carbonyl groups of both Leu199 and Leu215. The Trp carboxylate forms a salt bridge with Lys587 and hydrogen bonds to the side chains of both Gln500 and Ser523. Additionally, a water molecule links the Trp carboxylate to the side chains of Asp516 and Lys587.

5-Br-Trp binds to the same site but with a different binding pose (Figs. 5 ▸ *c* and 5 ▸ *d*). The indole moiety of 5-Br-Trp lies in the same plane as that of Trp but is flipped around an axis formed by C3 and C6, the positions of which are almost unchanged, while N1, C2, C5 and C7 are rotated by ∼180° around this axis. The indole moiety of 5-Br-Trp is surrounded by the same hydrophobic (Leu183, Leu196, Met216, Pro219, Phe373 and Phe524) and polar (Glu200 and Thr373) residues as that of Trp, but its NH group now forms a hydrogen bond to the carbonyl of Leu196 instead of Met216. The backbone and C^β^ atoms of 5-Br-Trp also move so that the polar interactions with the protein have to change. The side chain of Gln500, which binds both the amino and the carboxy group of Trp, rearranges and its amide group flips so that it can again form hydrogen bonds to both the amino and carboxy groups of 5-Br-Trp. The amino group of 5-Br-Trp now forms a hydrogen bond to the side chain of Asp516 instead of the carbonyl of Leu199. In addition, the 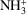 group of 5-Br-Trp forms a salt bridge with Asp516. The contacts of the carboxylate of the substrate are more conserved, including the hydrogen bond to the side chain of Ser523, the salt bridge to Lys587 and the hydrogen bond to Lys587 via a bridging water previously described for Trp.

**Figure 6:**
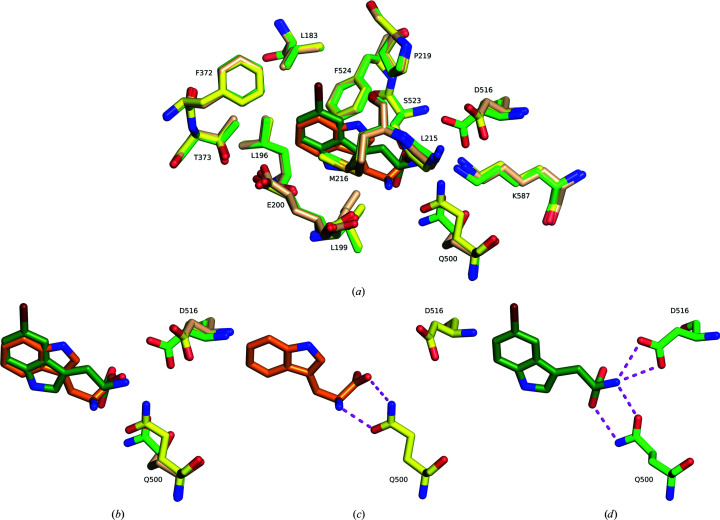
Structural changes in AetF upon substrate binding. Polar contacts are shown as magenta dashed lines. (*a*) Superposition of empty AetF (wheat C atoms), AetF–Trp (yellow C atoms, Trp in orange C atoms) and AetF–5-Br-Trp (green C atoms, 5-Br-Trp in dark green C atoms). Only Gln500 and Asp516 substantially change their conformation. (*b*) Upon binding Trp (orange C atoms), Gln500 (yellow C atoms) moves towards the ligand. Upon binding 5-Br-Trp (dark green C atoms), Gln500 adopts another rotamer and its side chain flips. Asp516 moves towards 5-Br-Trp and forms polar contacts that are not present in the binding of Trp. (*c*) Polar contacts of Trp with Gln500. (*d*) Polar contacts of 5-Br-Trp with Gln500 and Asp516.

**Figure 7:**
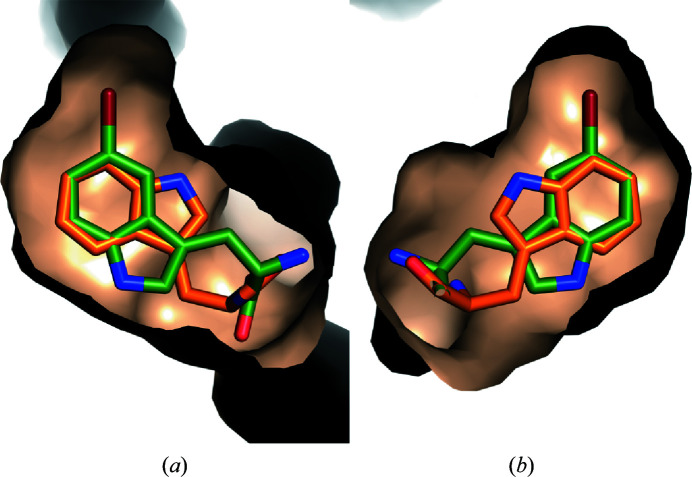
Surface representations of the substrate-binding site with bound Trp (orange C atoms) and 5-Br-Trp (green C atoms). The representations in (*a*) and (*b*) are rotated by 180°. The hydrophobic pocket surrounding the bromine is mostly formed by residues from the insertion in the NADP-binding domain (Leu183, Met217, Val220, Pro250 and Leu254) and by Phe372 from the FAD-binding domain.

A comparison of the substrate-binding sites in the structures without substrate, with Trp and with 5-Br-Trp reveals no major changes except for the movement of Gln500 and Asp516 (Fig. 6 ▸). The main factor determining the flipped binding pose of 5-Br-Trp appears to be the snug fit of bromine into a pre­formed hydrophobic pocket surrounded by Leu183, Met217, Val220, Pro250, Leu254 and Phe372 (Fig. 7 ▸). This pocket is empty in structures without substrate and in the complex with Trp.

### 3.5. The positioning of the halogenated C atom relative to Glu200 and Lys258 might explain the regioselectivity of AetF

**Figure 8:**
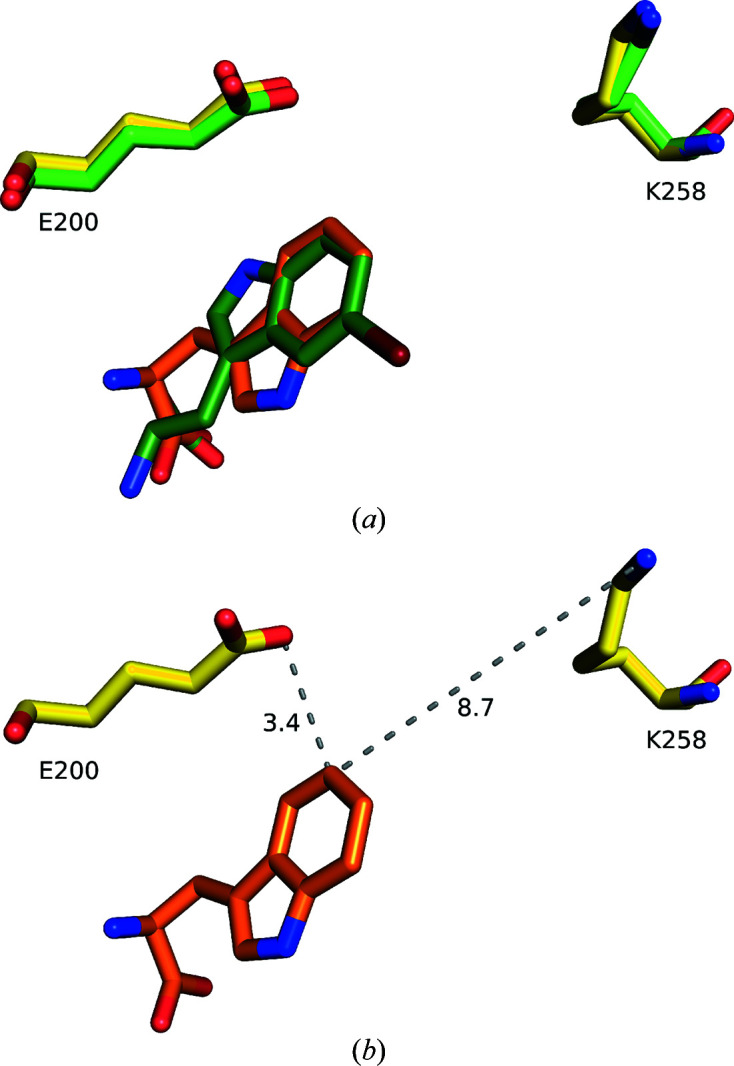
Positioning of the substrate relative to the catalytic Lys258 and the possibly catalytic Glu200. (*a*) Superposition of bound Trp (orange C atoms) and 5-Br-Trp (dark green C atoms) with Glu200 and Lys258 from the same structure in yellow and green, respectively. In each case the catalytically preferred C atom points towards the (possibly) catalytic amino acids. (*b*) Bound Trp (orange C atoms) from PDB entry 8cje chain *A*. Distances to the catalytically preferred position C5 are shown as grey dashed lines and are given in Å.

The flipped binding pose of 5-Br-Trp leads to an almost congruent position of the C atoms that will be halogenated in the two substrates. C5 of Trp and C7 of 5-Br-Trp are less than 0.5 Å apart when overlaying the two structures This suggests that C5 of Trp and C7 of 5-Br-Trp will be positioned productively relative to the catalytic residues (Fig. 8 ▸ *a*). The well studied two-component tryptophan halogenases presumably achieve regioselectivity by positioning the C atom to be halogenated with the shortest distance to the catalytic lysine (Dong *et al.*, 2005 ▸; Zhu *et al.*, 2009 ▸; Moritzer *et al.*, 2019 ▸). This is not the case in AetF. Averaged over all chains of the respective structures, the mean distance between the halogenated C atom and N^ɛ^ of Lys258 is 9.0 Å for C5 of Trp and 8.9 Å for C7 of 5-Br-Trp. In both structures the mean distance between C6 and N^ɛ^ of Lys258 is shorter, at 8.8 and 8.6 Å for Trp and 5-Br-Trp, respectively. However, Phe372 sterically blocks the C6 position, which might prevent halogenation of C6. Attempts to co-crystallize AetF with 6-Br-Trp yielded no crystals, while soaking AetF crystals with 6-Br-Trp led to empty active sites. This supports the assumption that a steric conflict with Phe372 might preclude the formation of 6-Br-Trp. The distance between the halogenated C atom and N^ɛ^ of the catalytic lysine is much longer in AetF (∼9 Å) than in the two-component tryptophan halogenases PrnA, RebH, PyrH and Thal (∼4 Å). However, distances similar to those in AetF are not unprecedented in two-component FDHs, with distances of 6.8 Å in the structure of a d-Trp–Gly dipeptide bound to Thal and of ∼7.5 Å in MalA′ (Schnepel *et al.*, 2023 ▸; Fraley *et al.*, 2017 ▸).

All two-component tryptophan halogenases contain a catalytic glutamate that is not conserved in FDHs that halogenate phenol or pyrrole (Andorfer & Lewis, 2018 ▸). The glutamate may function as a general base to abstract the proton from the Wheland intermediate, by stabilizing the positive charge of the Wheland intermediate or by polarizing the halogen of hypohalous acid (Dong *et al.*, 2005 ▸; Andorfer & Lewis, 2018 ▸; Flecks *et al.*, 2008 ▸). In AetF, Glu200, which is part of the indole-binding region, might perform an equivalent function. Thus, not only the distance to N^ɛ^ of Lys258 but also that to the carboxylate of Glu200 may determine the site selectivity. It is worth mentioning that for Trp, C5 has the shortest distance of all C atoms to the carboxylate of Glu200 (Fig. 8 ▸ *b*). For 5-Br-Trp, this distance is shortest for C7. At ∼3.5 Å, the distance between the halogenated C atom and the carboxylate of the possibly catalytic glutamate is almost identical in AetF and in the two-component FDHs PrnA, RebH, PyrH and Thal, hinting at a similar catalytic mechanism.

Our structures provide a clear-cut and simple explanation for the two successive regioselective brominations of Trp by AetF. The first step in the formation of 5,7-Br_2_-Trp by AetF is the conversion of Trp to 5-Br-Trp (Breinlinger *et al.*, 2021 ▸). We found that Trp and 5-Br-Trp bind to the substrate-binding site in different orientations. The flip of the indole moiety results in either C5 or C7 being positioned most closely to Glu200 and Lys258. Trp binds such that C5 is positioned correctly for halogenation. Addition of bromine to C5 increases the affinity of the flipped binding pose. We do not know whether 5-Br-Trp rearranges while bound to AetF or whether it dissociates from and rebinds to the enzyme. However, it is clear that at least a fraction of 5-Br-Trp has to emerge from AetF for further processing by the tryptophanase AetE to 5-Br-indole, which is an intermediate in AETX biosynthesis (Adak *et al.*, 2022 ▸).

The flip of the product upon the bromination of Trp on C5 by AetF differs from, for example, PrnA, which binds its product 7-Cl-Trp in the same orientation as the substrate Trp (Dong *et al.*, 2005 ▸). In PrnA, the chlorine of 7-Cl-Trp is thus very close to the N^ɛ^ atom of the catalytic lysine. Moreover, the strategy adopted by AetF for regioselective double halogenation clearly differs from those previously described for other enzymes. During the biosynthesis of kutzneride, the tryptophan 7-halogenase KtzQ and the tryptophan 6-halogenase KtzR act successively to produce 6,7-Cl_2_-Trp, with 7-Cl-Trp serving as a substrate for KtzR (Heemstra & Walsh, 2008 ▸). MalA, in contrast, regioselectively halogenates two positions of the substrate in the biosynthesis of malbranche­amides. There is no defined order as MalA produces roughly equal amounts of both singly halogenated intermediates during the reaction (Fraley *et al.*, 2017 ▸). Structures of the closely related FDH MalA′ showed the nonhalogenated educt, both singly halogenated intermediates and the doubly halogenated product to bind almost identically (Fraley *et al.*, 2017 ▸), suggesting that the product of the first halogenation does not need to reorient for the second halogenation to occur.

### 3.6. A tunnel connects the FAD and the distant tryptophan binding site

FMOs generally bind their substrates in the cleft between the large and the small dinucleotide-binding domains and in the direct vicinity of FAD (Romero *et al.*, 2018 ▸). While at least some Baeyer–Villiger monooxygenases from subgroup B1 can bind substrate and NADP at the same time (Yachnin *et al.*, 2012 ▸), in many FMOs of subgroup B2 the binding of substrate and NADP appear to be mutually exclusive, presumably because both occupy the same space (Eswaramoorthy *et al.*, 2006 ▸; Alfieri *et al.*, 2008 ▸; Cho *et al.*, 2011 ▸; Wang *et al.*, 2021 ▸). Accordingly, a previous attempt to dock the substrate Trp into an *AlphaFold*2 model of AetF used AetF in complex with FAD but lacking NADP because there was not sufficient space for both NADP and tryptophan to occupy the active site simultaneously (Jiang *et al.*, 2022 ▸). The docking resulted in several models with Trp placed next to the isoalloxazine ring of FAD, but even the top ten poses differed substantially from each other.

**Figure 9:**
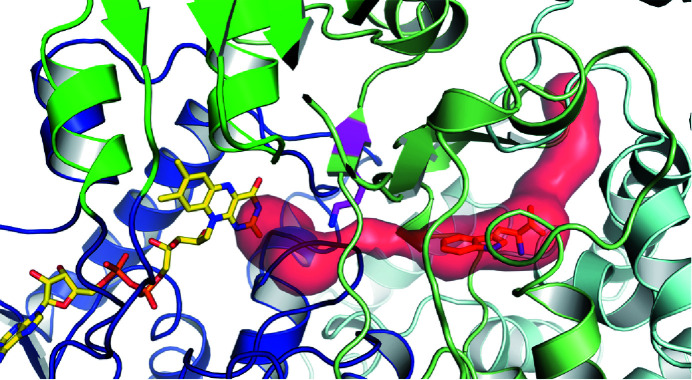
The tunnel connecting FAD and substrate. AetF is shown as a cartoon (domains coloured as in Fig. 2 ▸) in complex with FAD (yellow C atoms) and Trp (orange C atoms). *CAVER* 3.0 revealed a narrow tunnel (red surface) connecting FAD and Trp, which presumably allows diffusion of HO*X* from the cofactor to the substrate. The catalytic Lys258 is shown as sticks (magenta C atoms).

Our structures now reveal that the actual substrate-binding site is distant from the FAD-binding site and does not overlap with the presumed NADP-binding site. The C atom of the substrate that is to be halogenated and the reactive C4a of flavin are ∼16 Å apart, excluding direct reaction of flavin with the substrate. This is unusual for FMOs but analogous to the situation in two-component FDHs. In two-component tryptophan halogenases the substrate and flavin are connected by a tunnel of ∼10 Å in length (Dong *et al.*, 2005 ▸; Prakinee *et al.*, 2022 ▸). The separation of the redox-active flavin and the substrate is possible because the flavin C4a hydroperoxide intermediate reacts with a halide ion to generate hypohalous acid (HO*X*), which subsequently diffuses through the tunnel to the substrate. Analysis with *CAVER* 3.0 (Chovancova *et al.*, 2012 ▸; Pavelka *et al.*, 2016 ▸) shows that in AetF a tunnel of approximately 16 Å in length also connects the FAD and the substrate (Fig. 9 ▸). We propose that AetF uses a similar mechanism and the tunnel allows the directed diffusion of HO*X* to the substrate. The N^ɛ^ atom of the catalytic lysine of AetF (Lys258) points towards the tunnel and is located about halfway between the flavin C4a and the C atom of the substrate to be halogenated. Mutation of this lysine leads to a complete loss of halogenating activity, suggesting that it serves a role analogous to that of the catalytic lysine in two-component FDHs (Jiang *et al.*, 2022 ▸). We note, as a caveat, that the tunnel shown here has a radius of only 0.90 Å at the narrowest point. Thus, it would be too narrow to allow diffusion of HO*X*. The tunnel connecting FAD and tryptophan in the tryptophan 6-halogenase Thal also has several bottlenecks, with the narrowest having a radius of only 0.98 Å (Prakinee *et al.*, 2022 ▸). We assume that the side chains of Leu183 and Lys258, which have relatively large spatial freedom, can rearrange to widen the tunnel and thus allow the diffusion of HO*X*.

The tunnel in AetF further extends from the substrate to the surface, suggesting a route through which the substrate can access its binding site. Similar tunnels are present in ancestral FMO2, the protein in the PDB that has the highest structural similarity to AetF. The mammalian FMOs, on which the ancestral FMOs generated by ancestral sequence reconstruction are based, are membrane-binding enzymes and possess a tunnel system that presumably serves to guide multiple hydrophobic and hydrophilic substrates from the membrane or the cytosol towards the active site, where they can bind next to the flavin (Nicoll *et al.*, 2020 ▸). In an overlay of AetF and ancestral FMO2 (PDB entry 6sf0), Trp from AetF is located in close proximity to the bound detergent CYMAL6 that delineates the membrane–enzyme interface and sits at the start of a tunnel through which hydrophobic substrates are likely to diffuse to the active site (Supplementary Fig. S4; Nicoll *et al.*, 2020 ▸). The tunnel that provides access to the active site for various substrates in FMOs with broad substrate specificity may have evolved into a specific substrate-binding site in AetF.

### 3.7. Binding of 7-bromotryptophan to AetF opens a route to the biocatalytic production of various differentially dihalogenated tryptophans

**Figure 10:**
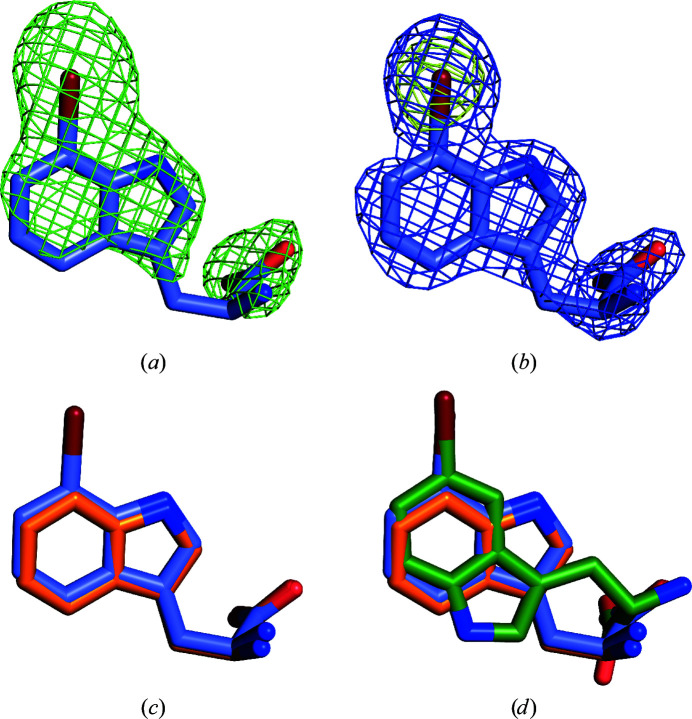
Binding of 7-Br-Trp. (*a*) Initial *mF*_o_ − *DF*_c_ density (green mesh, 3σ) before placing 7-Br-Trp. (*b*) Final 2*mF*_o_ − *DF*_c_ density (blue mesh, 1.5σ). The anomalous signal of bromine in 7-Br-Trp is shown as a yellow mesh (5σ). (*c*) 7-Br-Trp (blue C atoms) binds in the same pose as Trp (orange C atoms). (*d*) 5-Br-Trp (dark green C atoms) binds in a flipped orientation so that Br of 5-Br-Trp and 7-Br-Trp occupy the same space.

The different binding poses of Trp and 5-Br-Trp suggested to us that AetF should be able to bind 7-Br-Trp in the same orientation as Trp. Despite 7-Br-Trp not being a natural substrate of AetF, soaking AetF crystals with 7-Br-Trp provided highly interpretable difference densities after MR and anomalous difference density unambiguously showed the position of the Br atom (Figs. 10 ▸ *a* and 10 ▸ *b*). As predicted, 7-Br-Trp binds in the same binding pose as Trp (Fig. 10 ▸ *c*). The Br atom of 7-Br-Trp is located near the Br atom of 5-Br-Trp and occupies the previously described hydrophobic pocket (Fig. 10 ▸ *d*). C5 of 7-Br-Trp points towards the tunnel and the catalytic amino acids Glu200 and Lys258. Based on this structure, we predict that AetF might be able to halogenate 7-Br-Trp regioselectively at C5. This would open the way for site-specific differential double halogenation of Trp. Our structures suggest that AetF can accept both 5-Br-Trp and 7-Br-Trp as substrates for a second halogenation at positions 7 or 5, respectively. Enzymatic generation of 5-Br-Trp and 7-Br-Trp is possible with two-component FDHs such as PyrH (tryptophan 5-halogenase) and PrnA or RebH (tryptophan 7-halogenase). While PyrH, PrnA and RebH catalyze chlorination and bromination (Dong *et al.*, 2005 ▸; Yeh *et al.*, 2005 ▸; Zhu *et al.*, 2009 ▸), AetF can efficiently brominate and iodinate Trp (Jiang *et al.*, 2022 ▸). The successive use of two FDHs should therefore yield six different dihalogenated tryptophan derivatives with two different halogens, namely 5-*X*,7-*Y*-Trp, with *X* and *Y* being Cl, Br or I (Supplementary Fig. S5). Halogenated tryptophan can be further derivatized using chemical cross-coupling (Büchler *et al.*, 2019 ▸; Crowe *et al.*, 2021 ▸). The envisaged site-specific differential double halogenation of tryptophan may open new routes for subsequent chemical synthesis.

### 3.8. Homologous sequences containing the catalytic lysine could be single-component FDHs with different substrate specificities

FDHs have potential as biocatalysts for environmentally friendly halogenation reactions (Büchler *et al.*, 2019 ▸; Crowe *et al.*, 2021 ▸) and AetF has already been shown to be a versatile catalyst (Jiang *et al.*, 2022 ▸). Therefore, identifying new FDHs with altered substrate scope or halide specificity is of interest. Genome mining has already resulted in the identification of promising enzymes with new properties, but it has so far focused on two-component FDHs (Bayer *et al.*, 2013 ▸; Neubauer *et al.*, 2018 ▸; Gkotsi *et al.*, 2019 ▸). Moore and coworkers performed extensive sequence-similarity analysis for AetF and found that it is not related to two-component tryptophan halogenases, to the only other known single-component FDH Bmp5 or to any other characterized flavin-dependent monooxygenases, suggesting AetF to be unique among known halogenases (Adak *et al.*, 2022 ▸).

The presence of the catalytic lysine (Lys258 in AetF) might be a way to identify new halogenases among single-component FPMOs. While lysine at the position equivalent to Lys258 of AetF is not generally conserved among FMOs, it is conserved in the phenol halogenase Bmp5 (see above). In our hands, a pure sequence alignment did not result in correct alignment of the structurally equivalent catalytic lysines of AetF and Bmp5. Instead, the conservation becomes apparent in a structure comparison, for example an alignment of the *AlphaFold*2 model of Bmp5 with our structure of AetF. Other FMOs with a lysine in the same structural position as Lys258 of AetF might also be halogenases. To identify potential halogenases, we searched the AlphaFold/UniProt50 v.4 database (Bateman *et al.*, 2023 ▸) for structural homologs of AetF using *Foldseek* (van Kempen *et al.*, 2023 ▸). Among the top 50 hits (score ≥ 249; *E*-value ≤ 4.11 × 10^−8^), ten carry a lysine at the position equivalent to Lys258 of AetF (Supplementary Fig. S6). Thus, these ten hits might be novel single-component FDHs with an as yet unknown substrate specificity. If this prediction turns out to be true and substrates can be identified, these potential enzymes might become useful additions to the toolbox for biocatalytic halogenation.

## 4. Conclusion

The substrate-bound AetF structures nicely demonstrate that despite the high accuracy of *AlphaFold*2 protein models, experimental structures are still required. The actual substrate-binding site of AetF is distant from that anticipated based on analogy to known substrate-binding sites in other FMOs (Jiang *et al.*, 2022 ▸). Our structures facilitate a better mechanistic understanding of single-component FDHs in general and they may allow the identification of novel single-component FDHs based on structural conservation of the catalytic lysine. The Trp-bound and 5-Br-Trp-bound structures provide a clear explanation of the regioselective double bromination by AetF. Finally, our structures allow predictions of how to apply wild-type AetF for the generation of differentially double-halogenated tryptophan and they provide the necessary basis for structure-based modification of AetF to modify its regioselectivity and alter its substrate scope.

## 5. Related literature

The following references are cited in the supporting information for this article: Franceschini *et al.* (2012 ▸) and Valentino *et al.* (2020 ▸).

## Supplementary Material

PDB reference: AetF, 8cjd

PDB reference: complex with tryptophan, 8cje

PDB reference: complex with 5-bromotryptophan, 8cjf

PDB reference: complex with 7-bromotryptophan, 8cjg

Supplementary Tables and Figures. DOI: 10.1107/S2059798323004254/ag5042sup1.pdf

Raw data for PDB entry 8cjd.: https://doi.org/10.15785/SBGRID/1002

Raw data for PDB entry 8cje.: https://doi.org/10.15785/SBGRID/1003

Raw data for PDB entry 8cjf.: https://doi.org/10.15785/SBGRID/1004

Raw data for PDB entry 8cjg.: https://doi.org/10.15785/SBGRID/1005

Raw data for PDB entry 8cje.: https://doi.org/10.15151/ESRF-DC-1089735712

Raw data for PDB entry 8cjf.: https://doi.org/10.15151/ESRF-DC-1089719643

Raw data for PDB entry 8cjg.: https://doi.org/10.15151/ESRF-DC-1089739837

## Figures and Tables

**Table 1:** Data collection and processing Values in parentheses are for the outer shell.

Data set	Empty AetF	AetF–Trp	AetF–5-Br-Trp	AetF–7-Br-Trp
PDB code	8cjd	8cje	8cjf	8cjg
DOI of raw data (images)	10.15785/SBGRID/1002	10.15785/SBGRID/1003	10.15785/SBGRID/1004	10.15785/SBGRID/1005
		10.15151/ESRF-DC-1089735712	10.15151/ESRF-DC-1089719643	10.15151/ESRF-DC-1089739837
Diffraction source	P13, DESY	ID30B, ESRF	ID30B, ESRF	ID23-1, ESRF
Wavelength (Å)	0.9763	0.9184	0.9184	0.8856
Temperature (K)	100	100	100	100
Detector	EIGER X 16M	EIGER2 X 9M	EIGER2 X 9M	PILATUS 6M
Crystal-to-detector distance (mm)	252.160	183.01	211.07	320.81
Rotation range per image (°)	0.1	0.1	0.1	0.1
Total rotation range (°)	360	360	360	360
Exposure time per image (s)	0.008	0.02	0.02	0.02
Space group	*P*4_3_	*P*2_1_2_1_2_1_	*P*4_3_	*P*4_3_
*a*, *b*, *c* (Å)	123.79, 123.79, 88.00	87.27, 173.72, 173.96	122.70, 122.70, 87.42	122.33, 122.33, 87.16
α, β, γ (°)	90, 90, 90	90, 90, 90	90, 90, 90	90, 90, 90
Mosaicity (°)	0.036	0.065	0.081	0.051
Resolution range (Å)	50–1.70 (1.80–1.70)	50–1.80 (1.91–1.80)	50–1.90 (2.01–1.90)	50–2.30 (2.44–2.30)
Total No. of reflections	2043664 (313474)	3243964 (530909)	1391722 (228080)	795662 (121586)
No. of unique reflections	145683 (23109)	244069 (38986)	201422 (32268)	112302 (18128)
Completeness (%)	99.7 (98.4)	99.9 (99.5)	99.8 (98.8)	100 (99.9)
Multiplicity	14.03 (13.57)	13.29 (13.62)	6.91 (7.07)	7.09 (6.71)
〈*I*/σ(*I*)〉	16.19 (1.33)	10.19 (0.61)	6.55 (0.43)	7.97 (1.64)
CC_1/2_	0.999 (0.692)	0.998 (0.251)	0.996 (0.191)	0.988 (0.524)
*R*_meas_ (%)	9.0 (132.2)	19.3 (309.5)	19.3 (261.6)	24.8 (120.6)
Overall *B* from Wilson plot (Å^2^)	31.91	34.7	38.8	45.8

**Table 2:** Structure solution and refinement Values in parentheses are for the outer shell.

Data set	Empty AetF	AetF–Trp	AetF–5-Br-Trp	AetF–7-Br-Trp
PDB code	8cjd	8cje	8cjf	8cjg
Twin law		−*h*, −*l*, −*k*		−*k*, −*h*, −*l*
Twin fraction (%)		45		20
Resolution range (Å)	46.86–1.70 (1.72–1.70)	48.30–1.80 (1.83–1.80)	46.48–1.90 (1.92–1.90)	46.34–2.30 (2.34–2.30)
Completeness (%)	99.77 (97.79)	99.91 (93.70)	99.51 (91.74)	99.99 (95.03)
No. of reflections, working set	138236 (4523)	231613 (11326)	189995 (5866)	106642 (5375)
No. of reflections, test set	7215 (250)	12211 (598)	9993 (296)	5606 (281)
Final *R*_work_ (%)	15.90 (36.95)	16.03 (38.89)	18.31 (37.74)	15.32 (33.57)
Final *R*_free_ (%)	18.46 (39.88)	18.84 (40.95)	20.75 (39.96)	19.03 (39.56)
No. of non-H atoms				
Total	11930	22523	11469	11503
Protein	10634	20955	10669	10652
Ligand	163	272	228	196
Water	1165	1296	632	655
R.m.s. deviations				
Bond lengths (Å)	0.008	0.009	0.003	0.002
Angles (°)	0.85	0.97	0.58	0.53
Average *B* factors (Å^2^)				
Overall	38.47	30.43	46.28	28.25
Protein	37.96	30.39	46.45	28.38
Ligand	33.18	29.14	42.53	27.27
Water	43.76	31.36	44.42	26.39
Ramachandran plot				
Most favoured (%)	98.73	97.93	98.34	97.70
Allowed (%)	1.27	1.99	1.66	2.14
Outliers (%)	0	0.08	0	0.16
