# Supplementary material for: Structural basis of regioselective tryptophan dibromination by the single-component flavin-dependent halogenase AetF
Source: Acta Crystallogr D Struct Biol. 2023 Jun 14;79(Pt 7):596–609. doi: 10.1107/S2059798323004254 (PMC10306068; doi:10.1107/S2059798323004254)
Supplement: Supplementary file 1 [file d-79-00596-sup1.pdf]

STRUCTURAL
BIOLOGY

Volume 79 (2023)

Supporting information for article:

Structural basis of regioselective tryptophan dibromination by the single-component flavin-dependent halogenase AetF

Simon Gäfe and Hartmut H. Niemann

Table S1 Pairwise *DALI* alignment of AetF against the *AlphaFold2* model of Bmp5 (top row) and *DALI* hits for AetF against the PDB90.

The *DALI* run against the PDB was performed on 01.03.2023. The structure of Bmp5 used in the pairwise alignment is AF-U6BHD3-F1-model_v4.

No.	Protein	PDB	Z	rmsd	lali	nres	%id	reference
n.a.	flavin-dependent phenol brominase (Bmp5)	n.a.	27.6	3.5	438	515	16	(Agarwal *et al.*, 2014)
1	ancestral flavin-containing monooxygenase 2 (ancFMO2)	6sem	25.9	3.6	432	530	14	(Nicoll *et al.*, 2020)
2	ancestral flavin-containing monooxygenase 5 (ancFMO5)	6sek	25.6	3.1	426	504	15	(Nicoll *et al.*, 2020)
3	ancestral flavin-containing monooxygenase 3-6 (ancFMO3-6)	6se3	25.2	3.1	436	528	13	(Nicoll *et al.*, 2020)
4	ancestral flavin-containing monooxygenase 1 (ancFMO1)	7al4	20.8	3.2	437	524	14	(Bailleul *et al.*, 2021)
5	phenylacetone monooxygenase, a Baeyer-Villiger monooxygenase	1w4x	18.8	3.5	295	533	12	(Malito *et al.*, 2004)
6	protein with similarity to flavin-containing monooxygenases	1vqw	18.6	3.2	321	442	13	(Eswaramoorthy *et al.*, 2006)
7	pyrrolizidine alkaloid *N*-oxygenase (*Zv*PNO)	5nmx	18.3	3.3	321	409	13	(Kubitza *et al.*, 2018)
8	cyclohexanone monooxygenase	3gwd	18.2	3.6	301	531	13	(Mirza *et al.*, 2009)
9	steroid monooxygenase	4aos	18.2	3.5	297	532	14	(Franceschini *et al.*, 2012)
10	*S*-allyl-L-cysteine S-oxygenase	6wpu	17.9	3.2	332	439	14	(Valentino *et al.*, 2020)

Table S2 Boundaries of the FAD-binding and NADP-binding domains shown in Fig. 2

Protein	FAD-binding domain	NADP-binding domain	reference
AetF	1-132, 327-441	133-176, 294-326	this work
Bmp5	1-139, 324-432	140-199, 288-323	this work
AncFMO2	1-154, 331-442	155-213, 296-330	(Nicoll *et al.*, 2020)
*Zv*PNO[1)]	1-155 260-371	156-259	this work
	1-155, 260-425	156-259	(Kubitza *et al.*, 2018)

[1)] For *Zv*PNO the authors of the structure assigned the whole sequence either to the NADP- or to the FAD-binding domain. For Fig. 2, we defined the FAD-binding domain of *Zv*PNO based on structural elements conserved in the other three proteins. This leaves a short C-terminal extension (residues 372-425) as additional structural elements shown in light blue in Fig. 2.

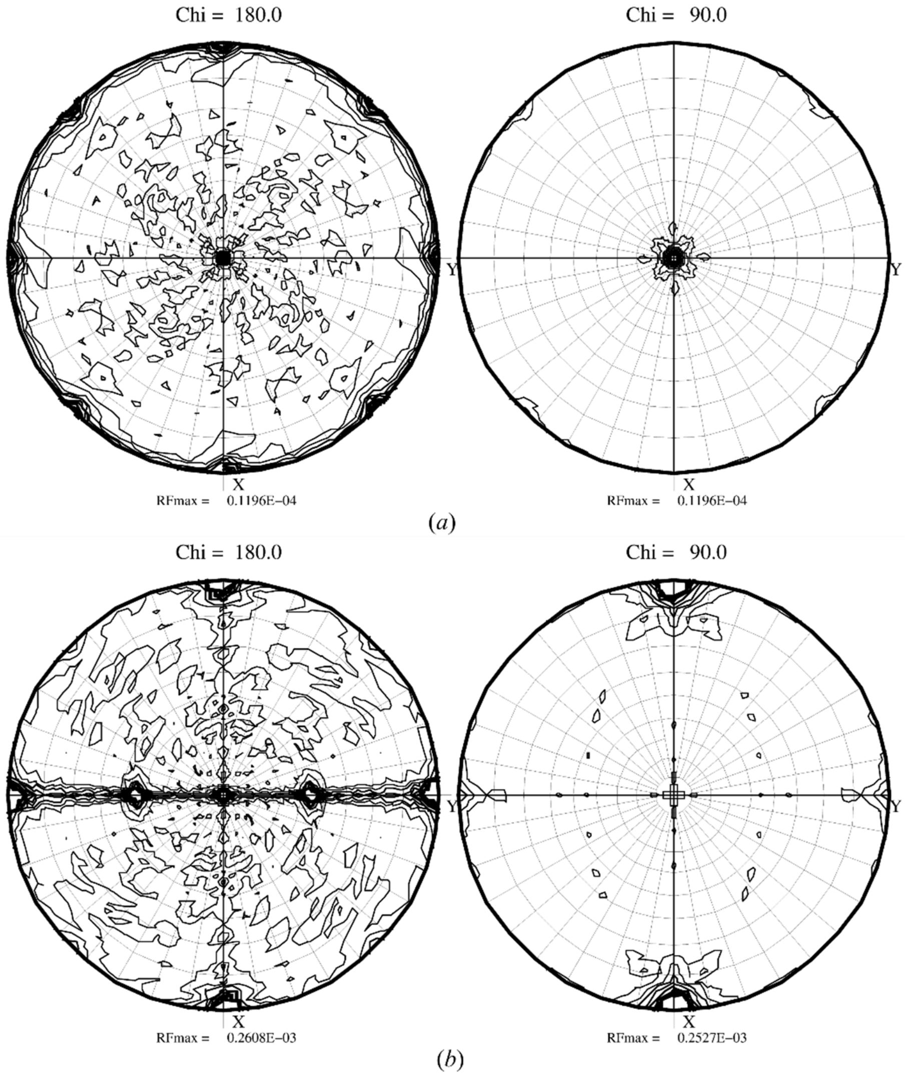

Figure S1 Self-rotation functions for χ=180 ° and χ=90 ° showing the location of 2-fold and 4-fold axes. (*a*) Untwinned $P4_3$ crystals (empty AetF). The short axis is the crystallographic 4-fold *c*-axis (*b*) Almost perfectly non-merohedrally twinned $P2_12_12_1$ crystals. The short axis is the *a*-axis. A non-crystallographic 4_3-axis lies parallel to *a* and the crystals are twinned by 90 ° rotation around *a*.

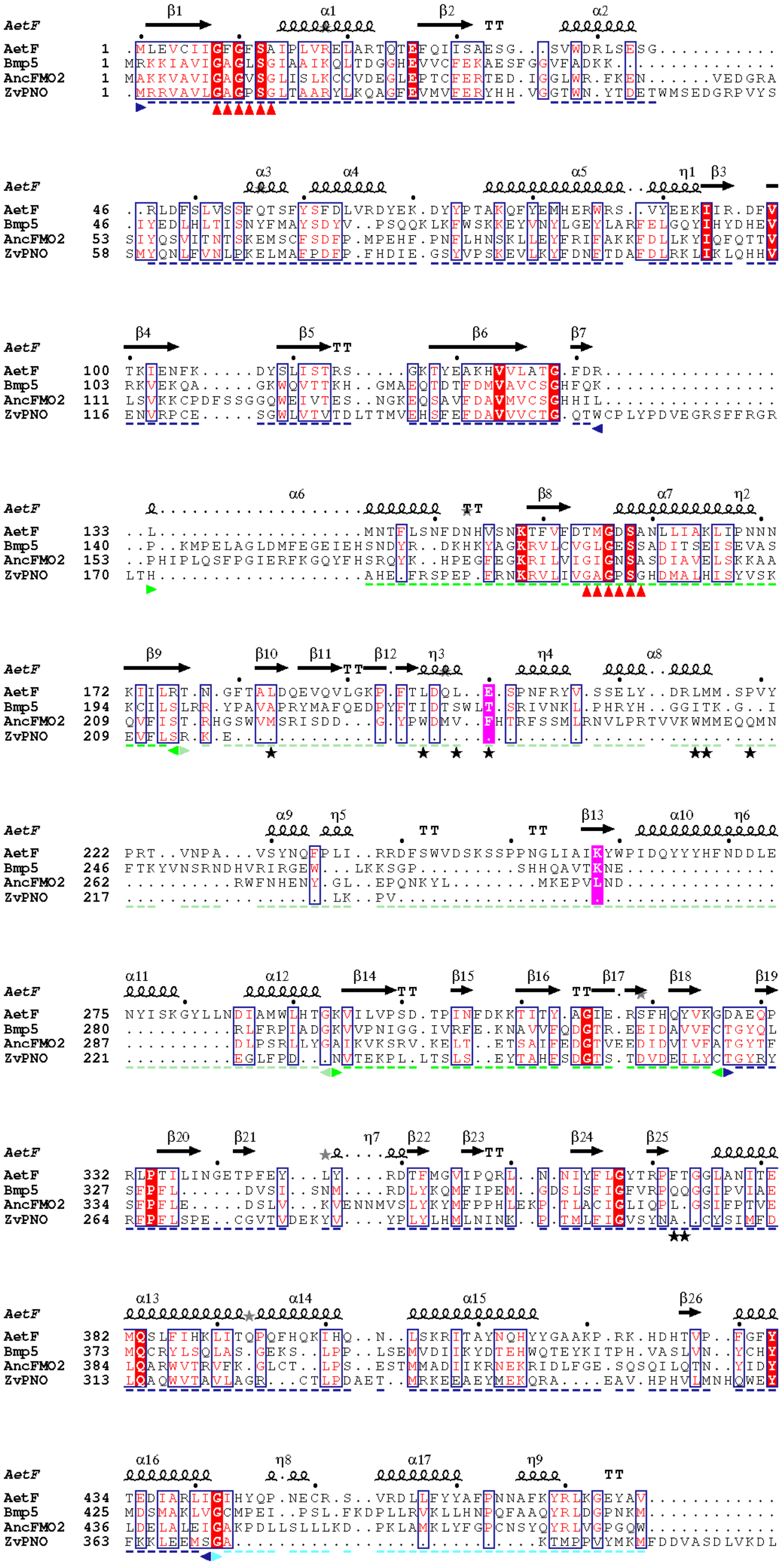
AetF
Bmp5
AncFMO2
ZvPNO

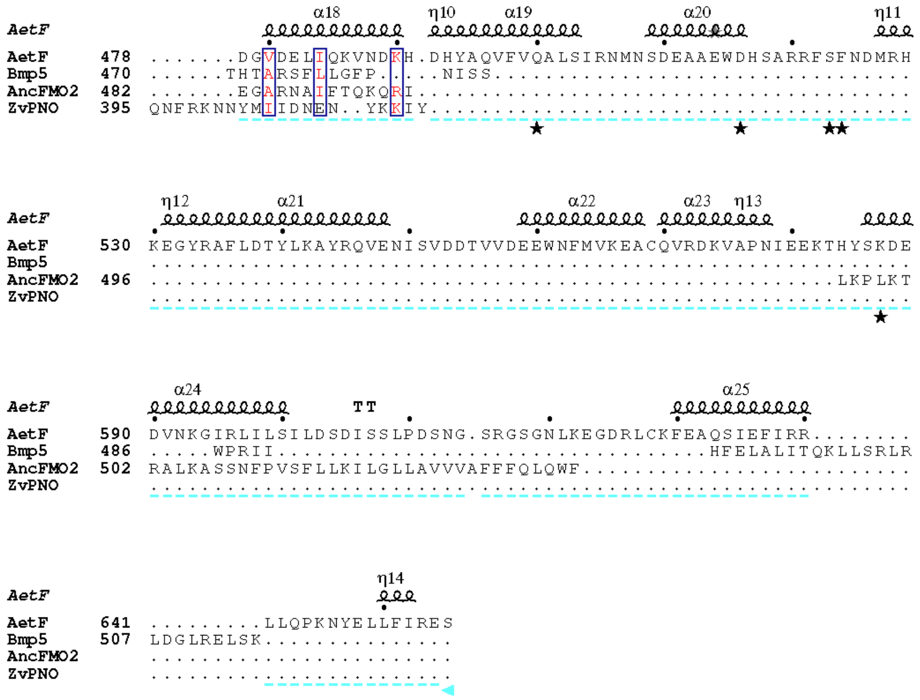

Figure S2 Pairwise *DALI* alignment of AetF against Bmp5 (AF-U6BHD3-F1-model_v4), AncFMO2 (6sf0 *B*) and *Zv*PNO (5nmx *A*). Missing structural elements were copied from the respective *AlphaFold2* models. Domains in AetF are marked by dashed lines and domain borders by arrows (same color code as in Fig. 2). Consensus sequences for dinucleotide binding (GXGXXG and GXGXXA) are marked by red triangles and residues involved in binding of Trp or 5-Br-Trp are indicated by black stars. The catalytic amino acids found in AetF (E200 and K258) are highlighted in magenta.

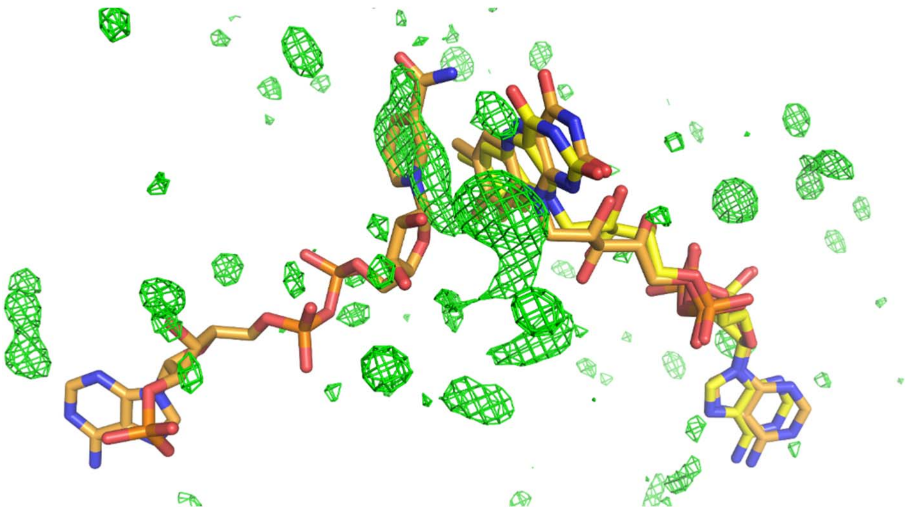

Figure S3 Difference density (mF_o-DF_c density, 3 σ, green mesh) at the expected location of the NADP-binding site. The larger contiguous difference density near the FAD in AetF (yellow carbon atoms) could be interpreted by a nicotinamide riboside. For comparison, NADP and FAD from 6sf0 (light orange carbon atoms) are shown (structures superimposed on the FADs). The NADP in AetF would need to adopt a different binding pose compared to 6sf0 to fit the shown difference density.

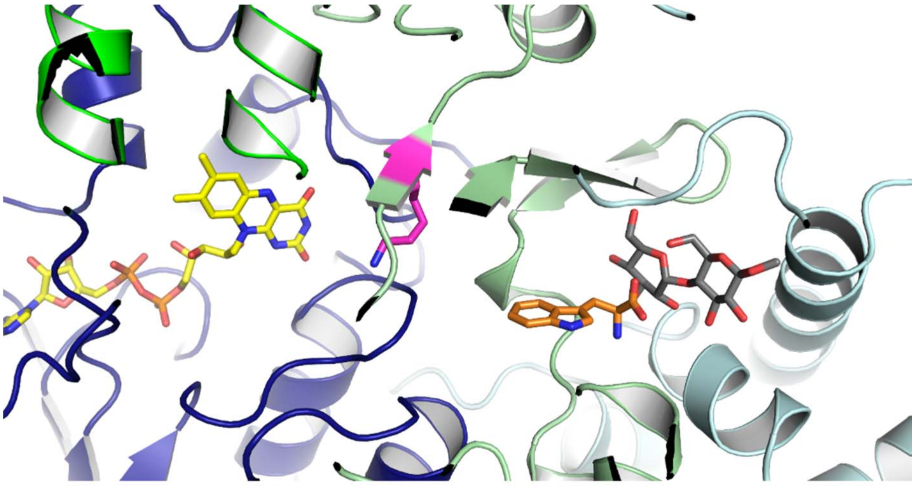

Figure S4 Comparison of Trp binding in AetF and CYMAL6 in AncFMO2. Our structure for AetF–Trp (cartoon, domains coloured as in Fig. 2) and AncFMO2 (6sf0 *D*) were superimposed at the FADs. Both Trp (orange carbon atoms) and CYMAL6 (grey carbon atoms) bind to a similar site in the respective enzyme.

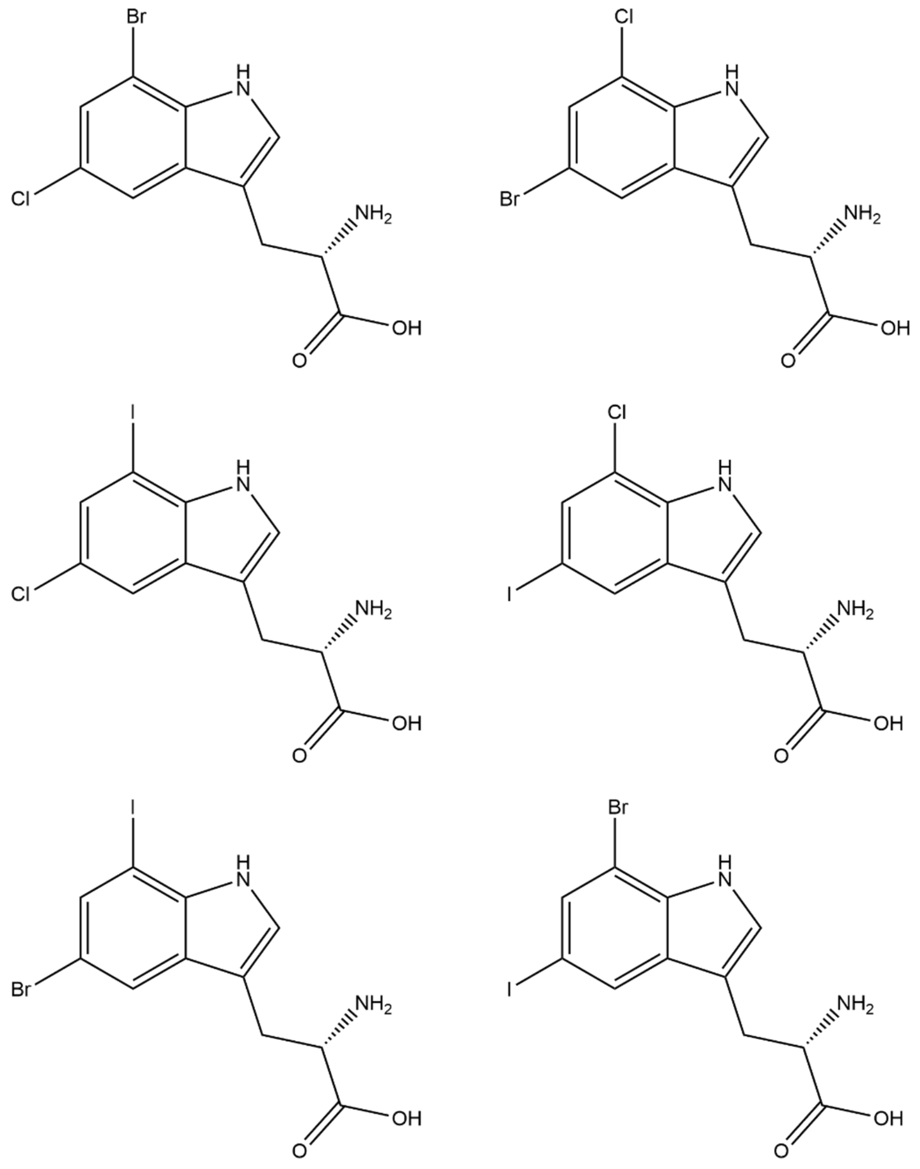

Figure S5 Possible products of tryptophan halogenation with two different halogens. With prior use of a suitable two-component halogenase, a regioselective second halogenation by AetF should be possible. This would allow site-specific differential double halogenation of Trp.

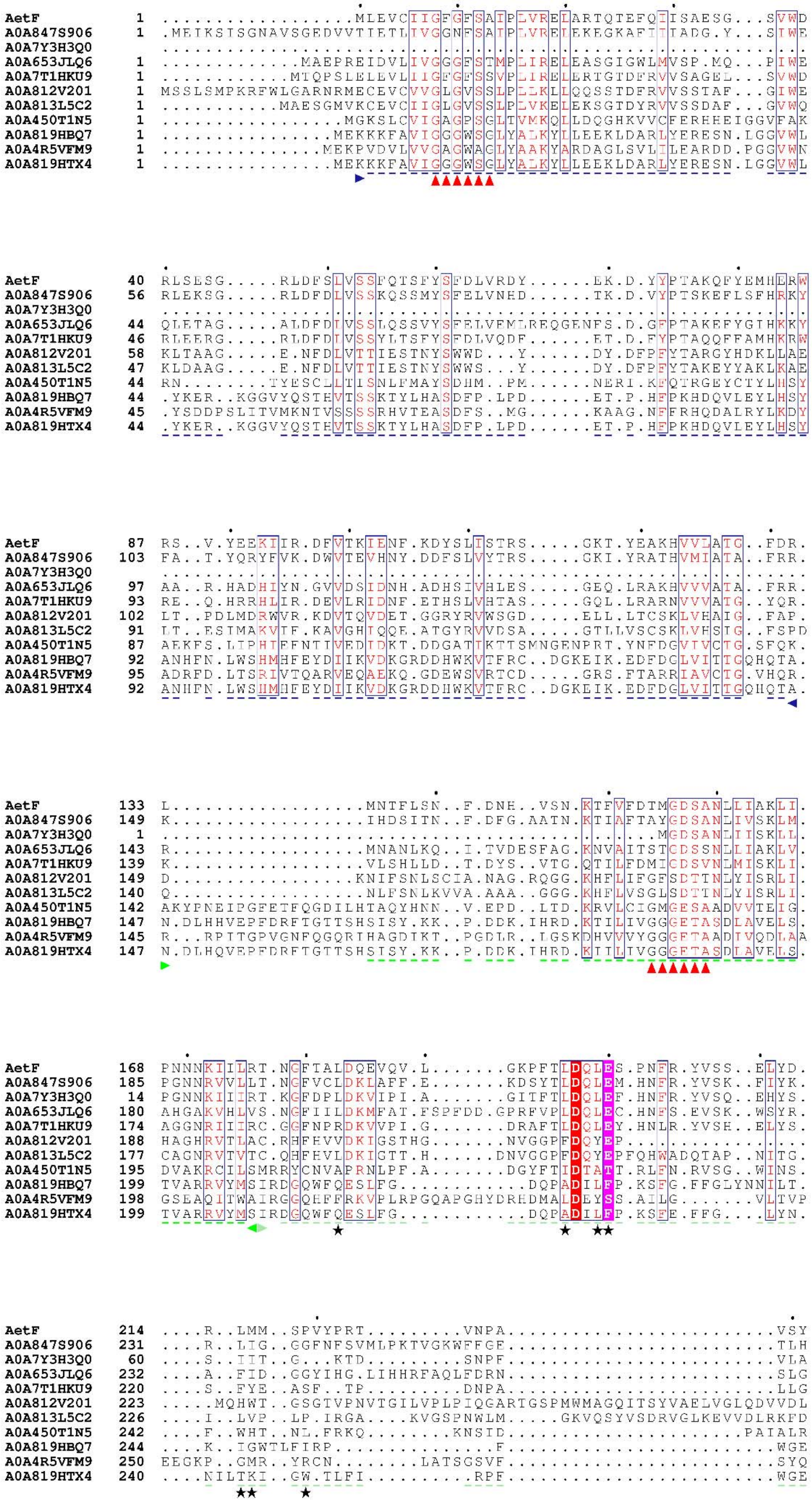
AetF 1 ..................MLEVCIIGFGFSAIPLVRELARTQTEFQIISAESG...SVWD
A0A847S906 1 .MEIKSISGNAVSGEDVVTIETLIVGGNFSAIPLVRELEKEGKAFIIIADG.Y...SIWE
A0A7Y3H3Q0 ....
A0A653JLQ6 1 .............MAEPREIDVLIVGGGFSTMPLIRELEASGIGWLMVSP.MQ...PIWE
A0A7T1HKU9 1 ............MTQPSLELEVLIIGFGFSSVPLIRELERTGTDFRVVSAGEL...SVWD
A0A812V201 1 MSSLSMPKRFWLGARNRMECEVCVVGLGVSSLPLLKLLQQSSTDFRVVSSTAF...GIWE
A0A813L5C2 1 ...........MAESGMVKCEVCIIGLGVSSLPLVKELEKSGTDYRVVSSDAF...GVWQ
A0A450T1N5 1 .................MGKSLCVIGAGPSGLTVMKQLLDQGHKVVCFERHHEIGGVFAK
A0A819HBQ7 1 ................MEKKKFAVIGGGWSGLYALKYLLEEKLDARLYERESN.LGGVWL
A0A4R5VFM9 1 ...............MEKPVDVLVVGAGWACLYAAKYARDAGLSVLILEARDD.PGGVWN
A0A819HTX4 1 ................MEKKKFAVIGGGWSGLYALKYLLEEKLDARLYERESN.LGGVWL

AetF 40 RLSESG.....RLDFSLVSSFQTSFYSFDLVRDY......EK.D.YYPTAKQFYEMHERW
A0A847S906 56 RLEKSG.....RLDFDLVSSKQSSMYSFELVNHD......TK.D.VYPTSKEFLSFHRKY
A0A7Y3H3Q0 ....
A0A653JLQ6 44 QLETAG.....ALDFDLVSSLQSSVYSFELVEMLREQGENFS.D.GFPTAKEFYGTHKKY
A0A7T1HKU9 46 RLEERG.....RLDFDLVSSYLTSFYSFDLVQDF......ET.D.FYPTAQQFFAMHKRW
A0A812V201 58 KLTAAG.....E.NFDLVTTIESTNYSWWD...Y......DY.DFPFYTARGYHDKLLAE
A0A813L5C2 47 KLDAAG.....E.NFDLVTTIESTNYSWWS...Y......DY.DFPFYTAKEYYAKLKAE
A0A450T1N5 44 RN........TYESCLLTISNLFMAYSDHM..PM......NERI.KFQTRGEYCTYLHSY
A0A819HBQ7 44 .YKER..KGGVYQSTHVTSSKTYLHASDFP.LPD......ET.P.HFPKHDQVLEYLHSY
A0A4R5VFM9 45 .YSDDPSLITVMKNTVSSSSRHVTEASDFS..MG......KAAG.NFFRHQDALRYLKDY
A0A819HTX4 44 .YKER..KGGVYQSTHVTSSKTYLHASDFP.LPD......ET.P.HFPKHDQVLEYLHSY

AetF 87 RS..V.YEEKIIR.DFVTKIENF.KDYSLISTRS.....GKT.YEAKHVVLATG..FDR.
A0A847S906 103 FA..T.YQRYFVK.DWVTEVHNY.DDFSLVYTRS.....GKI.YRATHVMIATA..FRR.
A0A7Y3H3Q0 ....
A0A653JLQ6 97 AA..R.HADHIYN.GVVDSIDNH.ADHSIVHLES.....GEQ.LRAKHVVVATA..FRR.
A0A7T1HKU9 93 RE..Q.HRRHLIR.DEVLRIDNF.ETHSLVHTAS.....GQL.LRARNVVVATG..YQR.
A0A812V201 102 LT..PDLMDRWVR.KDVTQVDET.GGRYRVWSGD.....ELL.LTCSKLVHAIG..FAP.
A0A813L5C2 91 LT..ESIMAKVTF.KAVGHTQQE.ATGYRVVDSA.....GTLLVSCSKLVHSIG..FSPD
A0A450T1N5 87 AEKFS.LIPHTFFNTIVEDTDKT.DDGATTKTTSMNGENPRT.YNFDGVTVCTG.SFQK.
A0A819HBQ7 92 ANHFN.LWSHMHFEYDIIKVDKGRDDHWKVTFRC..DGKEIK.EDFDGLVITTGQHQTA.
A0A4R5VFM9 95 ADRFD.LTSRIVTQARVEQAEKQ.GDEWSVRTCD.....GRS.FTARRIAVCTG.VHQR.
A0A819HTX4 92 ANHFN.LWSHMHFEYDIIKVDKGRDDHWKVTFRC..DGKEIK.EDFDGLVITTGQHQTA.

AetF 133 L..................MNTFLSN..F.DNH..VSN.KTFVFDTMGDSANLLIAKLI.
A0A847S906 149 K..................IHDSITN..F.DFG.AATN.KTIAFTAYGDSANLIVSKLM.
A0A7Y3H3Q0 1 ....MGDSANLIISKLL.
A0A653JLQ6 143 R..................MNANLKQ..I.TVDESFAG.KNVAITSTGDSSNLLIAKLV.
A0A7T1HKU9 139 K..................VLSHLLD..T.DYS..VTG.QTILFDMIGDSVNLMISKLI.
A0A812V201 149 D.................KNIFSNLSCIA.NAG.RQGG.KHFLIFGFSDTTNLYISRLI.
A0A813L5C2 140 Q..................NLFSNLKVVA.AAA..GGG.KHFLVSGLSDTTNLYISRLI.
A0A450T1N5 142 AKYPNEIPGFETFQGDILHTAQYHNN..V.EPD..LTD.KRVLCIGMGESAADVVTEIG.
A0A819HBQ7 147 N.DLHHVEPFDRFTGTTSHSISY.KK..P.DDK.IHRD.KTILIVGGGETASDLAVELS.
A0A4R5VFM9 145 R...RPITGPVGNFQGQRIHAGDIKT..PGDLR..LGSKDHVVVYGGGFTAADIVQDLAA
A0A819HTX4 147 N.DLHQVEPFDRFTGTTSHSISY.KK..P.DDK.IHRD.KTILIVGGGETASDLAVELS.

AetF 168 PNNNKIILRT.NGFTALDQEVQV.L.......GKPFTLDQLES.PNFR.YVSS..ELYD.
A0A847S906 185 PGNNRVVLLT.NGFVCLDKLAFF.E.......KDSYTLDQLEM.HNFR.YVSK..FIYK.
A0A7Y3H3Q0 14 PGNNKIIIRT.KGFDPLDKVIPI.A.......GITFTLDQLEF.HNFR.YVSQ..EHYS.
A0A653JLQ6 180 AHGAKVHLVS.NGFIILDKMFAT.FSPFDD.GPRFVPLDQLEC.HNFS.EVSK..WSYR.
A0A7T1HKU9 174 AGGNRIIIRC.GGFNPRDKVVPI.G.......DRAFTLDQLEY.HNLR.YVSH..ELYS.
A0A812V201 188 HAGHRVTLAC.RHFHVVDKIGSTHG.......NVGGPFDQYEP.................
A0A813L5C2 177 CAGNRVTVTC.QHFHVLDKIGTT.H......DNVGGPFDQYEPFQHWADQTAP..NITG.
A0A450T1N5 195 DVAKRCILSMRRYCNVAPRNLPF.A.......DGYFTIDTATT.RLFN.RVSG..WINS.
A0A819HBQ7 199 TVARRVYMSIRDGQWFQESLFG............DQPADILFP.KSFG.FFGLYNNILT.
A0A4R5VFM9 198 GSEAQITWAIRGGQHFFRKVPLRPGQAPGHYDRHDMALDEYSS.AILG.......VLTVP
A0A819HTX4 199 TVARRVYMSIRDGQWFQESLFG............DQPADILFP.KSFE.FFG....LYN.

AetF 214 ....R..LMM..SPVYPRT.........VNPA.........................VSY
A0A847S906 231 ....R..LIG..GGFNFSVMLPKTVGKWFFGE.........................TLH
A0A7Y3H3Q0 60 ....S..IIT..G...KTD.........SNPF.........................VLA
A0A653JLQ6 232 ....A..FID..GGYIHG.LIHHRFAQLFDRN.........................SLG
A0A7T1HKU9 220 ....S..FYE..ASF..TP.........DNPA.........................LLG
A0A812V201 223 .....MQHWT..GSGTVPNVTGILVPLPIQGARTGSPMWMAGQITSYVAELVGLQDVVDL
A0A813L5C2 226 ....I..LVP..LP.IRGA....KVGSPNWLM.....GKVQSYVSDRVGLKEVVDLRKFD
A0A450T1N5 242 ....F..WHT..NL.FRKQ........KNSID......................PAIALR
A0A819HBQ7 244 ....K..IGWTLFIRP...............F.........................WGE
A0A4R5VFM9 250 EEGKP..GMR..YRCN........LATSGSVF.........................SYQ
A0A819HTX4 240 ....NILTKI..GW.TLFI..........RPF.........................WGE

```
AetF        232  N..Q.....F.PLI..R..R...D..FSWV..DSKSSPPNGL..IAIKYWPIDQYYYHFN
A0A847S906  258  V..K.....Y.PMA..N..RNWKL..AKELGWDATSPVPNGY..IVIKYWPIDAYQRLYD
A0A7Y3H3Q0   75  T..Q.....F.PSS..V..R...D..MSHI..TTKSTPSSGT..IVIKYWPVDLYAKEFG
A0A653JLQ6  258  V..R.....H.PKS..I..R...PHKDIRHFFKAKAPVENGH..IAIKYWPIDVYKLYCE
A0A7T1HKU9  236  S..Q.....F.PAT..V..R...D..NRKA..GAAASPHSGS..VATKYWPVDAYSASFG
A0A812V201  276  R..QFDTEYQ.EAGRGL..V...N..PA.....NVDVPGGLG..YLVKQWPVDEYARFYD
A0A813L5C2  268  T..E.....VQYCA..QGLV...D..PAKT.......EVPGGLGLLVKQWPVDEYAKYYN
A0A450T1N5  263  G..E.....W....CLK..A...G..N..........EPNQ..VITKNE..........
A0A819HBQ7  258  GGTG.....I.AEW..K..P...R..C..........QLFHG..FINKSR..........
A0A4R5VFM9  273  .GHG.....V.AEW..V..N...D..IP.........WFRQ..FFNKNG..........
A0A819HTX4  258  GGTG.....I.AEW..K..P...R..C..........QLFHG..FINKSR..........
```

```
AetF        271  D..D..LENYISKGYLLNDIAMWLHTGKVILVPS.D.TPINFDKKTITY..AGIERSFHQ
A0A847S906  302  D.GQ..LEQHIEDGYLMNDIGYFVEQGKVDLWSKSQ.TTIDTDTRTIKW..GDKIVQYDA
A0A7Y3H3Q0  114  D..N..LEENIAKGYLLNDIAMWIKTGKVIVVPP.E.SKIDFDKKTISY..GGIERSFYQ
A0A653JLQ6  301  E..T..LEQKIADGYLINDLPFFLEHGHVKLWDKAA.TTIDHEAMTLSE..DGENASFDM
A0A7T1HKU9  275  H..R..LEEAIEEGYLLNDIALWIHTGRVVVVPR.E.TPVDLEARVVHH..DGLEIPFDH
A0A812V201  319  D..DRYRDWMLKEGVYLNDTYFFTQQGLVSLHPR.SDVEHI.AEKRYRV..RGEEVEFDE
A0A813L5C2  307  DPSF..QEWMLKSGVYLNDIYFFMQQGLVKVHKR.DEVQHL.AGKRYLV..AGEEVEFDE
A0A450T1N5  282  ...................RIFNSIIDGKVIPNTV.G.IEKIT.ENSVLF.KDGVQEEIDT
A0A819HBQ7  281  ...................GVVNKVASGMVIPKRQ.I.NSIK..EQLVTFDNEEKPVHIDH
A0A4R5VFM9  295  ...................HALEHVWNQRVTPARG.I.QACD..GRTVTF.EDGRTCQATH
A0A819HTX4  281  ...................GVVNKVASGMVIPKRQ.I.NSIK..EQLVTFDNKEKPVHIDH
```

```
AetF        323  YVKGDAEQPRLPTILING....ETPFE..Y.LYRDTFMGVIPQRLN.NIYFLGYTRPFTG
A0A847S906  356  LIEGDREMPNLPLIISKRSGSMDKPYE..Y.VYRDNFMGIAPRELK.NIYLVGYTRPTSG
A0A7Y3H3Q0  166  YVTGDAEKPKLPPIMING....DTPFE..Y.KYRENFMGVIPKKLN.NVYLIGYTRPMTG
A0A653JLQ6  354  IIDGDQEIPAVPEIRAH.GESGVEVFA..Y.KPREQFMGVTSPGLR.NIYTIGFTRPFTG
A0A7T1HKU9  327  RFSGDAEQPRLPPILIDG....TTPYT..Y.RYRDNFMGVLPKDLQ.RIQGP........
A0A812V201  373  VLNCDEAKHKTLSMPF........CPN..Y.SYTDHLFGIWSRKHP.NLYFLGTTRPYTG
A0A813L5C2  361  VLGCCEAKHKLLEVRG........CRP..F.TYSEHLYGIWNRIHP.NLFFLGTTRPYTG
A0A450T1N5  320  IMCCTGFRMAFPFLK........NEYS..FTNPRLLYKHMFHPELGKKIVFAGFVRPQQG
A0A819HBQ7  319  ILFCTGYRWFHSFFN........DNFRKKT.KIECLYQLIFPVNLP.NLAFVGSTRPVFG
A0A4R5VFM9  332  VICCFGYEGDVSFLP........EELR.RV.PTDKLYRLVFHPDDP.SLSFFGFARPTIL
A0A819HTX4  319  ILFCTGYRWFHSFFD........DNFRKET.KIECLYQLIFPVNLP.NLAFVGSTRPVFG
                                                                        **
```

```
AetF        375  GLANITEMQSLFIHKLITQPQFHQKIHQ..N..LSKRITAYNQHYYGA.AKPRKHDHTVP
A0A847S906  412  GLNNIIEMQCLFVHRMITDKSFNDSIYQ..N..IEERIKQYDIEYYGT.AEKRLTDHLVF
A0A7Y3H3Q0  218  GVANISEMQSIFTHKLITQPNFLRDIRY..N..LEERIDNYNKHYYGS.TPPGKTDHSVY
A0A653JLQ6  409  GLNNITEMQCLLVHRMIADPDFRNSIRT..N..IDQRIADYNATYYTK.RPEKKTDHLVW
A0A7T1HKU9  371  .........AAHP...............................................
A0A812V201  421  AFGCIAEVNAMFVHRMITESKFHGQLVR..Q..FPGLMHSQRVSHYVQ.ATEPDKMHVHW
A0A813L5C2  409  AFGCVSELSSLFVHRMLTNSSFQDFISK..Q..FPSLMHRQRVTHHAS.VSDPDKLHVQW
A0A450T1N5  370  GVPVIAEMQGRYLALLY.NDDV..SLPPEDQ..LPALIQADKESEEREFYITPYVTSLVN
A0A819HBQ7  369  SIPSLAELQARRVAQVFS.GRDC..LPS..PTNMSIWLQKYWKRHAQLYPFEGRLQQLVN
A0A4R5VFM9  381  SLPYMIELQCLLAERVW.SGAL..ELPDREA..RYAEAEADAASLDAFFGYERSNRNITC
A0A819HTX4  369  SIPSLAELQARRVAQVFS.GRDC..LPS..PTNMSIWLQKYWKRHAQLYPYEGHLQQLVN
```

```
AetF        430  FGFYTEDIARLIGIHY....QPNE.....CR...S.VRDLLFYYAFPNNAFKYRLKGEY.
A0A847S906  467  YGFYTHDLAKLIGIAG....SAFT.....GK...S.LKDIFKYYLFPNNAFKYRQEGKY.
A0A7Y3H3Q0  273  YGFYTDDIARLMGIDF....KPKE.....CT...K.MKDLVFYYAFPNNAFKYRLRGEY.
A0A653JLQ6  464  YGTFTDEVAKLLDIRP....ARAD.....MP...G.KKGLMRYYMFPNNVFRFREKGRY.
A0A7T1HKU9  375  ..........LSRA..............................................
A0A812V201  476  AGMHCLWLSRVLGCDI....TFSE....AFN...R.G.MVLEWMTGPINALRCRICGPY.
A0A813L5C2  464  AGMHCLRIAKTLGCDL....SYRE....AGR...L.G.LRVEQQTGPINALRCRITGPY.
A0A450T1N5  425  YSHYMESLGRLIGCSP....E.VS.....WF...KDFKLASKIWLGPHFAIQYRLNGPQ.
A0A819HBQ7  424  QFEYSDFLANQLKVKP....RLWK.....LFFSQP.QKWYTIYFQSPWTPFLFRMNAIET
A0A4R5VFM9  436  PFWYLTLVMGALGKEKTGLRALFRRFNPFNN...W.QEFSRII.RVPLSPFLIRLL..V.
A0A819HTX4  424  QFEYSDFLANQLKVKP....RLWK....SLL...I.K.......................
```

```
AetF        476  .AVD..GVDELIQKVNDKHDH.YAQV..............FV.QAL.SIR.N..M.....
A0A847S906  513  .KVD..GVKEMVEKIWQEHSG.FSLM..............KH.YIL.NYL.L..M.....
A0A7Y3H3Q0  319  .AVE..GIDKVVEKINKQYKD.FMAI..............FA.YVL.TSNTR..N.....
A0A653JLQ6  510  .AVD..GVDQLIEHTSKQYHD.YKVL..............AL.LVI.RYP.F..F.....
A0A7T1HKU9       ............................................................
A0A812V201  522  .AVP..GACDKYKASCQKIG..TSYM..............FL..................
A0A813L5C2  510  .ALP..GAAARYKRSCKKIN..TSFM..............SL.NMI.YRN.W..GDRMVV
A0A450T1N5  471  .AFKEEDQKEIRHFLRTFP....LYF..............PKSTYA.LLI.G........
A0A819HBQ7  474  NDEK..LAYSRHLKCI.PRRDQ......................................
A0A4R5VFM9  488  .SDHGPDARLRMEEYI.....SPMPFGFRRDPGASLLRYVM.FYVLAIG.VPRL.....
A0A819HTX4       ............................................................
                                                           *
```

```
AetF             ............................................................
A0A847S906       ............................................................
A0A7Y3H3Q0       ............................................................
A0A653JLQ6       ............................................................
A0A7T1HKU9       ............................................................
A0A812V201       ............................................................
A0A813L5C2  546  VSWFVALCCSAGFAWLKMSEVEKLAFYAVFLYLLTKATLLSQWRGVVLMLAFDNLYHKRA
A0A450T1N5       ............................................................
A0A819HBQ7       ............................................................
A0A4R5VFM9       ............................................................
A0A819HTX4       ............................................................

AetF        508  .......................................N....................
A0A847S906  545  ............QVNILAFICLLPMSVFIKVPLSLLFLYN....................
A0A7Y3H3Q0  352  .......................................M....................
A0A653JLQ6  542  .......................................ELLALATILLAPVPWWVKIPA
A0A7T1HKU9       ............................................................
A0A812V201       ............................................................
A0A813L5C2  606  WIMLLSNVCQLVFNCICFLHGTTYDVPLLVRLGLPVDVIF....................
A0A450T1N5       ............................................................
A0A819HBQ7       ............................................................
A0A4R5VFM9  534  .......................................L....................
A0A819HTX4       ............................................................

AetF        509  .......SDEAAEWDHSA..........................................
A0A847S906  573  .......PFIVLVVALAT.................NLHRYLNLFIVCAMVATCFYPGPLI
A0A7Y3H3Q0  353  .......GEDRSDWLKQQ..........................................
A0A653JLQ6  563  AIIHNRLPFTSTLVGKFGLPTRESKAIFNYRKAISFPVLAYPLVAAATWAAAGTDAAFAF
A0A7T1HKU9       ............................................................
A0A812V201       ............................................................
A0A813L5C2  646  .......LVLGRISY.PR..........................................
A0A450T1N5       ............................................................
A0A819HBQ7       ............................................................
A0A4R5VFM9  535  .......AI..DTLFD............................................
A0A819HTX4       ............................................................

AetF        520  ....................RRFSFNDMRHKEG..YRAFLD.TYLKAYRQVENI......
A0A847S906  609  PGLSLLAVFVTIFIGRKAGITRIPFNDLKNKSK..YHDFFK.RYSEAFNRVAERKKSTAV
A0A7Y3H3Q0  364  ....................KRAFFNDMRPKDA..YNSFVE.KYFKAFRKVKNL......
A0A653JLQ6  623  SAGLLAYVYAMIHLGTAKGWNRKFFCDMKSKRSPEMVRFFE.KYRAAFGRVKGTRALNET
A0A7T1HKU9  379  .............................HPALHRRPGQHRRDAEPAGAQADRQRGVPAA
A0A812V201       ............................................................
A0A813L5C2  656  ....................AFF..GDMRVRKP..YAKWLFNTYLP..............
A0A450T1N5       ............................................................
A0A819HBQ7       ............................................................
A0A4R5VFM9       ............................................................
A0A819HTX4       ............................................................

AetF        551  ........SVDDTVVDE..EWNFMVKEACQVRDKVAPNIEEKTHYSKDEDVNKGIRLILS
A0A847S906  666  ANAVLTEE....................................................
A0A7Y3H3Q0  395  .......NQVED.IFDE..EWNQLVKIAGKTRDEVIKETEDLGTPKWSEEIHAAADLVRS
A0A653JLQ6  682  P...........................................................
A0A7T1HKU9  410  HHRQSGGADRRLQPPLL..R........................................
A0A812V201       ............................................................
A0A813L5C2  678  .........RSAKFTSGGP.........................................
A0A450T1N5       ............................................................
A0A819HBQ7       ............................................................
A0A4R5VFM9  542  .........................RIARRRIARQ.........................
A0A819HTX4       ............................................................

AetF        601  ILDSDISSLPDSNGSRGSGNLKEGDRLCKFEAQSIEFIRRLLQPKN............YE
A0A847S906       ............................................................
A0A7Y3H3Q0  445  LAVN...DLGA...........ISDQSIEKFHEHFKLLASMKDPQE............YD
A0A653JLQ6       ............................................................
A0A7T1HKU9       ............................................................
A0A812V201  545  ......................................NMIYRTWG............DR
A0A813L5C2       ............................................................
A0A450T1N5  502  ........................................GWILGTLFPFIQKFRKRP..
A0A819HBQ7       ............................................................
A0A4R5VFM9  552  ...........................GRSLAPRPAAMEFARAASS............PP
A0A819HTX4       ............................................................
```

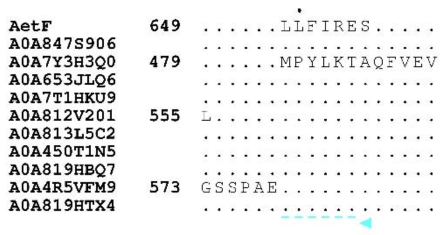

Figure S6 (Including previous pages) Pairwise *DALI* alignment of AetF against *AlphaFold2* models of the top ten hits containing a lysine at the position equivalent to the catalytic lysine in AetF (K258) found in a *Foldseek* search against the *AlphaFold/Uniprot50 v4 database*. The missing loop (609 – 628) in AetF (8cjd *A*) was copied from the *AlphaFold2* model. Domains in AetF are marked by dashed lines and domain borders by arrows (same color code as in Fig. 2). Consensus sequences for dinucleotide binding (GXGXXG and GXGXXA) are marked by red triangles and residues involved in the binding of Trp or 5-Br-Trp are indicated by black stars. The catalytic amino acids found in AetF (E200 and K258) are highlighted in magenta.
